# Improved Methods for Fluorescence Microscopy Detection of Macromolecules at the Axon Initial Segment

**DOI:** 10.3389/fncel.2016.00005

**Published:** 2016-02-16

**Authors:** Musaad A. Alshammari, Tahani K. Alshammari, Fernanda Laezza

**Affiliations:** ^1^Graduate Studies Abroad Program, King Saud UniversityRiyadh, Saudi Arabia; ^2^Department of Pharmacology and Toxicology, University of Texas Medical BranchGalveston, TX, USA; ^3^Mitchell Center for Neurodegenerative Diseases, University of Texas Medical BranchGalveston, TX, USA; ^4^Center for Addiction Research, University of Texas Medical BranchGalveston, TX, USA; ^5^Center for Biomedical Engineering, University of Texas Medical BranchGalveston, TX, USA

**Keywords:** FGF14, axon initial segment, nodes of Ranvier, immunohistochemistry, sodium channel, Nav1.6, Ankyrin-G

## Abstract

The axonal initial segment (AIS) is the subcellular compartment required for initiation of the action potential in neurons. Scaffolding and regulatory proteins at the AIS cluster with ion channels ensuring the integrity of electrical signaling. Interference with the configuration of this protein network can lead to profound effects on neuronal polarity, excitability, cell-to-cell connectivity and brain circuit plasticity. As such, the ability to visualize AIS components with precision provides an invaluable opportunity for parsing out key molecular determinants of neuronal function. Fluorescence-based immunolabeling is a sensitive method for morphological and molecular characterization of fine structures in neurons. Yet, even when combined with confocal microscopy, detection of AIS elements with immunofluorescence has been limited by the loss of antigenicity caused by fixative materials. This technical barrier has posed significant limitations in detecting AIS components alone or in combination with other markers. Here, we designed improved protocols targeted to confocal immunofluorescence detection of the AIS marker fibroblast growth factor 14 (FGF14) in combination with the cytoskeletal-associated protein Ankyrin-G, the scaffolding protein βIV-spectrin, voltage-gated Na^+^ (Nav) channels (especially the Nav1.6 isoform) and critical cell type-specific neuronal markers such as parvalbumin, calbindin, and NeuN in the mouse brain. Notably, we demonstrate that intracardiac perfusion of animals with a commercially available solution containing 1% formaldehyde and 0.5% methanol, followed by brief fixation with cold acetone is an optimal and sensitive protocol for FGF14 and other AIS marker detection that guarantees excellent tissue integrity. With variations in the procedure, we also significantly improved the detection of Nav1.6, a Nav isoform known for its fixative-sensitivity. Overall, this study provides an ensemble of immunohistochemical recipes that permit excellent staining of otherwise invisible molecules within well-preserved tissue architecture. While improving the specific investigation of AIS physiology and cell biology, our thorough study can also serve as a roadmap for optimizing immunodetection of other fixative-sensitive proteins expanding the repertoire of enabling methods for brain studies.

## Introduction

The axon initial segment (AIS) is a specialized subcellular compartment that commences a short distance from the neuronal soma just past the axon hillock (Duflocq et al., 2011). The highly organized structure of the enriched mesh of ion channels and accessory proteins at the AIS is required for generation of the action potential (Buffington and Rasband, 2011; Duflocq et al., 2011; Yoshimura and Rasband, 2014; Akin et al., 2015; Papandreou et al., 2015). Cytoskeletal-associated, scaffolding and regulatory proteins such as Ankyrin-G, βIV-spectrin, and FGF14 (Ogawa and Rasband, 2008; Duflocq et al., 2011; Xiao et al., 2013) are localized at the AIS where they cluster with voltage-gated Na^+^ (Nav) channels (and other channels) ensuring the integrity of electrical signaling. Interference with this protein network can lead to deficits in excitability and eventually neuronal death (Hsu et al., 2014). Not surprisingly, GWAS studies reporting associations between mutations, copy variants or SNPs in genes coding for AIS proteins and neuropsychiatry disorders are rapidly emerging in the field of brain disorders (Hsu et al., 2014). Thus, enabling technologies and methodologies to probe for expression, pattern distribution and localization of AIS molecules would provide a powerful tool for investigating the biology of complex brain disorders and designing novel therapeutics (Schafer et al., 2009; Hsu et al., 2014).

Immunohistochemistry (IHC) coupled to fluorescence labeling is one of the most routinely established approaches in neuroscience and general biology laboratories. Fluorescence-based immunolabeling typically employs a sequence of procedures including exposing the cells/tissue specimen to primary unlabeled antibodies against specific epitopes (the antigen determinant of the protein of interest), followed by fluorophor-conjugated secondary antibodies. This can provide simultaneous multi-channel visualization of proteins in cells and tissues through fluorescence microscopy (epifluorescence, confocal, multiphoton, super-resolution, etc., Fritschy, 2008). In its basic form in fixed preparations, IHC allows one to profile the expression level and pattern distribution of given analytes across developmental stages with cell type- and brain area-specific precision in complex animal models and human specimens (Breunig et al., 2007; Evers et al., 2010). If used in live tissue, immunolabeling can also provide time course information on protein trafficking and targeting in native conditions (Progatzky et al., 2013; Mottillo et al., 2014). When applied to fixed tissue specimens, optimal results in IHC depend greatly on two basic elements: optimal fixation and tissue preservation (Schneider Gasser et al., 2006). Of the main fixatives generally employed in IHC (glutaraldehyde, paraformaldehyde, methanol/acetone), paraformaldehyde at various concentrations is widely used as it provides the most straightforward and rapid fixative method to expose antibody epitope(s) without compromising cell/tissue morphology (Bocksteins et al., 2012). However, detecting proteins in fine sub-cellular structures could be challenging using conventional fixatives (Schneider Gasser et al., 2006; Lorincz and Nusser, 2008b, 2010; Christensen et al., 2014). As a tight matrix of intracellular proteins, the AIS forms a detergent-resistant membrane microdomain that can mask epitopes and reduce antibody accessibility (Lorincz and Nusser, 2008a; Galiano et al., 2012; Gutzmann et al., 2014; Stradleigh and Ishida, 2015). Support for this hypothesis comes from early successes in AIS protein IHC that employied detergent extraction methods, unconventional permeabilization (Garrido et al., 2001, 2003), whole-brain immersion (Rasband et al., 1999), and ultra-brief nearly without fixative (Shakkottai et al., 2009; Shavkunov et al., 2013; Xiao et al., 2013; Bosch et al., 2014; Tian et al., 2014). Yet, each of these methods depend heavily on the experimenter, lead to poorly reproducible results between laboratories and do not guarantee well-preserved cell and tissue morphology. In addition, they are sub-optimal for labeling with other markers unrelated to the AIS. Dual labeling of AIS proteins with other neuronal markers has been achieved combining standard IHC with mRNA *in situ* hybridization (for AIS markers) (Brackenbury et al., 2010; Han et al., 2012; Verret et al., 2012; Lorenzo et al., 2014), but this approach is much less powerful for investigating protein biology.

To develop a sensitive, accurate and reproducible method for immunolabeling of AIS markers that could be broadly applicable to multichannel fluorescence microscopy, we have exhaustively screened fixation recipes under ten different experimental conditions. We initially focused on optimizing our protocol for fluorescence-based IHC of FGF14, a member of the AIS that has been proven especially problematic to study. We then extended our methods especially to Nav1.6, a critical Nav channel isoform in the brain circuit and preferential binding partner of FGF14. As a result, we present an optimal protocol for detection of FGF14 (Wang et al., 2002; Lou et al., 2005; Laezza et al., 2007, 2009; Xiao et al., 2007, 2013; Shakkottai et al., 2009; Shavkunov et al., 2013; Hsu et al., 2014; Bosch et al., 2015; Tempia et al., 2015) and other fixative-sensitive proteins that warrant high quality detection of AIS molecules alone or in combination with cell type-specific neuronal markers. We expect that our protocol will have a broad impact on the neuroscience community allowing reproducible and reliable detection of proteins that have been otherwise undetectable.

## Materials and methods

### Animals

*Fgf14*^+∕+^ and *Fgf14*^−∕−^ (control for FGF14 staining) mice were maintained on an inbred C57/BL6J background (greater than ten generations of backcrossing to C57/BL6J). Animals were bred in the UTMB animal care facility by mating heterozygous *Fgf14*^+∕−^ males and females. The University of Texas Medical Branch operates in compliance with the United States Department of Agriculture Animal Welfare Act, the Guide for the Care and Use of Laboratory Animals, and IACUC approved protocols. Mice were housed, *n* ≤ 5 per cage, with food and water *ad libitum*. All genotypes described were confirmed by PCR analysis conducted by our lab and/or Charles River Laboratories International.

### Preparation of brain sections

Frozen tissue (Table 1): Adult mice were deeply anesthetized with isoflurane (Baxter, Deerfield, IL) administered in a small chamber. Whole brains were then removed, frozen in liquid nitrogen, and stored at −80°C until use. In preparation for sectioning, brains were embedded in OCT compound (Tissue-Tek®, Ted Pella, Inc., Redding, CA) and 10–15 μm-thick sagittal or coronal sequential brain sections were prepared at −18 to −20°C using a Leica CM1850 cryostat (Leica Microsystems, Buffalo Grove, IL). Sections were then mounted on glass microscope slides (Fisherbrand® Superfrost Plus, Fisher Scientific, Pittsburgh, PA) and stored at −80°C. Before staining, Frozen sections were immersed in 1%, 4% PFA, acetone alone, acetone followed by methanol, or 2% PFA + 0.2% Glutaraldehyde fixative solutions. Fixed mouse brain (Table 2): Adult mice were first deeply anesthetized with 2,2,2-tribromoethanol (250 mg/kg i.p.; Sigma-Aldrich, Saint Louis, MO), then briefly perfused (intra-cardiac; flow rate: 8–10 ml/min for 2–5 min) with cold 1X phosphate buffer (PBS, pH = 7.4), followed by 10 min of cold 1%, 4% paraformaldehyde (Sigma-Aldrich, catalog number 441244), 1% formaldehyde containing 0.5% methanol (a dilution of 37% formaldehyde solution in PBS, MasterTech Scientific, catalog number fxfor37gal), or Optimal Fix™ (an alcohol-based tissue fixative that contains <50% ethanol, <50% ethanediol, <1% Zinc-chloride; MasterTech Scientific, catalog number fxoptgal); 1% and 4% paraformaldehyde solutions were freshly prepared by gradually dissolving paraformaldehyde (wt/vol) in warm dH_2_O (50–60°C) containing in final concentration 5N NaOH, 5M NaCl, and 0.2M of a mixture of sodium phosphate monobasic and dibasic (MSP/DSP). All solutions were adjusted to a pH of 7.4 and stored at 4°C. In preparation for sectioning and to ensure complete tissue fixation, brains were removed carefully and post-fixed in either: 1% paraformaldehyde, 1% formaldehyde + 0.5% methanol, Optimal Fix™ for 1 h, 4% paraformaldehyde for 1 h, or 24 h at 4°C and then cryopreserved in 20–30% sucrose/PBS at 4°C. Brains were allowed to completely sink to the bottom of the container before sectioning. Brains were embedded in OCT compound (Tissue-Tek®, Ted Pella, Inc.) and sectioned sagittally into 15–25 μm-thick slices at −18 to −20°C using a Leica CM1850 cryostat (Leica Microsystems). Floating brain sections (4% paraformaldehyde post-fixed for 24 h) were stored in a cryoprotectant solution (ethylene glycol based; 30% ethylene glycol, 30% glycerol, 10% 0.2 M sodium phosphate buffer pH 7.4, in dH_2_O) at −20°C; glass slide-mounted brain section (fixed with 1% formaldehyde contains 0.5% methanol, Optimal Fix™, or 1%, 4% paraformaldehyde post-fixed for 1 h) were stored in −80°C. For 4% PFA direct immersion tissue preparations were as described previously in Rasband et al. (1999). Briefly, brains were extracted, washed with cold 1X PBS and immersed in cold 4% PFA solution (pH ~ 7.2) for 30 min. Following incubation in 20–30% sucrose, brains were let to sink to the bottom of the container. Then, 10–15 μm thick sagittal sections were collected and mounted on glass microscope slides and stored at −80°C (for more details see Table 2, Supplementary Figures 6, 7). Acute brain slices (Table 3): Adult mice were anesthetized with isoflurane (Baxter) in a small chamber, then decapitated, and coronal brain slices of 300–400 μm were cut with a vibratome VT1200S (Leica, Buffalo Grove, IL) in ice-cold regular artificial CSF solution containing (in mM 125 NaCl, 2.5 KCl, 2 MgCl_2_, 2.5 CaCl_2_, 1.25 NaH_2_PO_4_, 26 NaHCO_3_, and 20 glucose with 95% O_2_ and 5% CO_2_ bubbled). Then slices were transferred to a recovery chamber with 95% O_2_ and 5% CO_2_ bubbled regular artificial CSF containing 25 μM Dimethyl sulfoxide (DMSO) at room temperature for 2 h. Next, slices were transferred to a 24-well plate (Greiner Bio One, North Carolina) and rinsed with cold 1X PBS, then incubated for 30 min in a 1% formaldehyde + 0.5% methanol solution (MasterTech Scientific) at 4°C, followed by 1-day immersion in 20–30% sucrose/PBS in preparation for sectioning. For sectioning, coronal slices were embedded in OCT compound and sectioned at 20–25 μm using a CM1850 Leica cryostat then mounted on glass slides (Fisher Scientific) and stored at −80°C until further use.

**Table 1:**
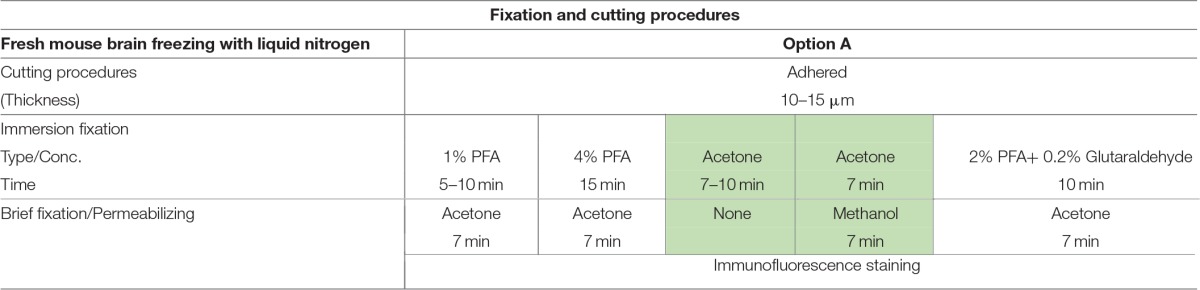
**Fixation protocols used in Option A (fresh-frozen tissue preparation)**.

*Optimal protocols for AIS components detection are highlighted with green. Acetone ± MeOH methods were reproduced with two mice per group (Fgf14^+∕+^ and Fgf14^−∕−^), ≤ 4 sections per mouse. PFA, Paraformaldehyde*.

**Table 2:**
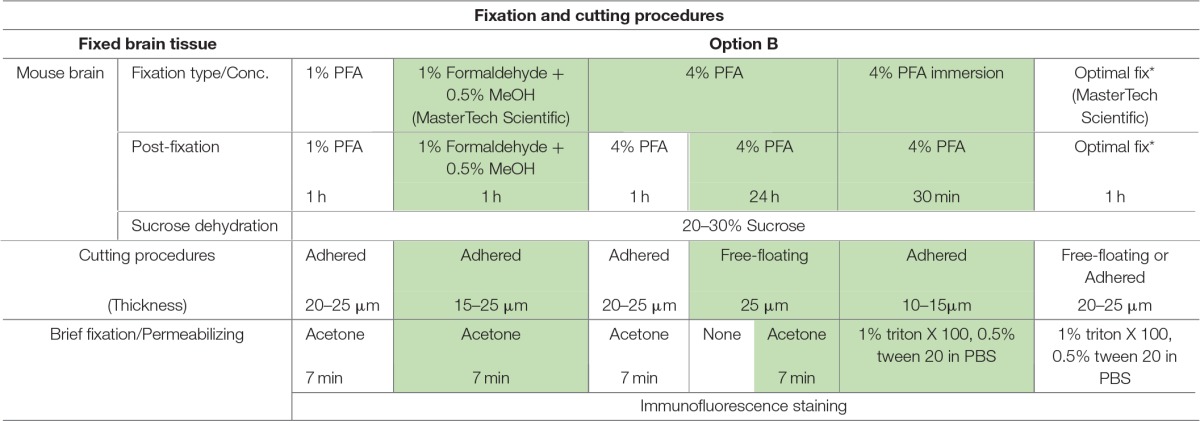
**Fixation protocols used in Option B (perfused fixed tissue preparation)**.

Optimal protocols for AIS components detection are highlighted with green. 4% PFA + acetone method was reproduced with four mice per group (Fgf14^+∕+^ and Fgf14^−∕−^), ≤ 3 sections per mouse. 1% Formaldehyde + 0.5% MeOH method was reproduced with five mice per group (Fgf14^+∕+^ and Fgf14^−∕−^), ≤ 3 sections per mouse. 4% PFA immersion method was reproduced with two mice, ≤ 4 sections per mouse. PFA, Paraformaldehyde, MeOH, Methanol, PBS, Phosphate-buffered solution, ^*^Optimal fix™: Alcohol based tissue fixative that contains 50% Ethanol, 50% Ethanediol, 1% Zinc-chloride (MasterTech Scientific).

**Table 3:**
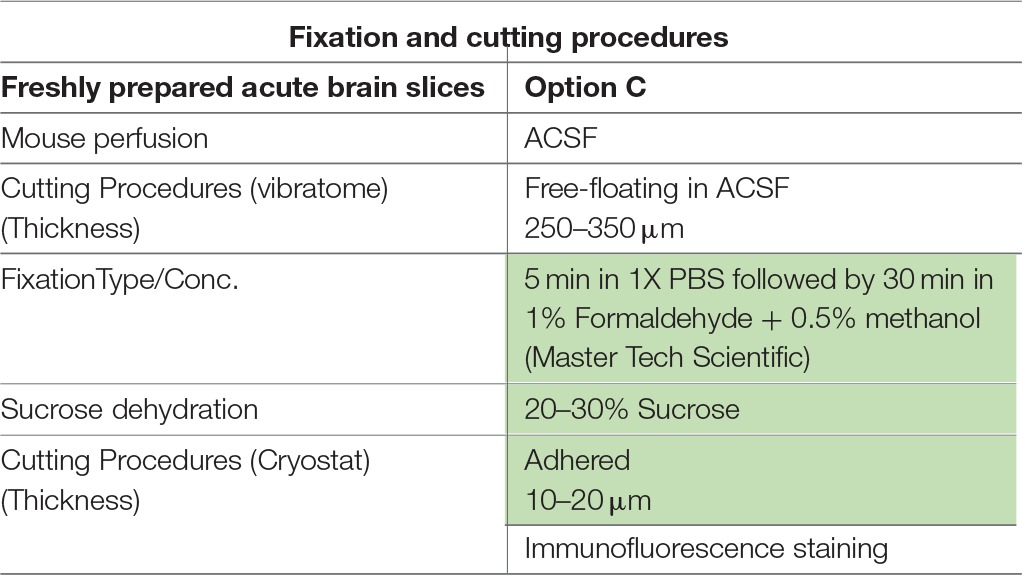
**Fixation protocols used in Option C (acute brain slice preparation)**.

*Optimal protocols for AIS components detection are highlighted with green. 30 min 1% Formaldehyde + 0.5% MeOH method was reproduced with two mice, ≤ 3 sections per mouse. ACSF, Artificial cerebrospinal fluid; PBS, Phosphate-buffered solution*.

### Immunofluorescence

A schematic representation of all IHC steps can be found in **Scheme 2**. Day 1: free floating or glass slide-mounted sections were washed with 1X Phosphate buffer PBS or Tris-buffered saline TBS, then pre-incubated with permeabilizing agent (1% Triton X-100, 0.5% Tween-20 in PBS; −20°C acetone and/or −20°C methanol). Then, slices were washed extensively with 1X PBS or 1X TBS and incubated with a blocking buffer consisting of 10% normal goat serum NGS (Sigma-Aldrich), 5% donkey serum DS (Santa Cruz Biotechnology, Dallas, TX) or a mixture of 5% NGS and 3% DS in 1X TBS containing 0.3% Triton X-100 for 1 h. This was followed by overnight incubation at 4°C with primary antibodies in 3% bovine serum albumin (BSA; Sigma-Aldrich) and 1X PBS containing 0.1% Tween-20. Primary antibodies used in this study were: mouse anti-FGF14 (1:300, NeuroMab, catalog number 75-096); IgG2a mouse anti-Ankyrin-G (1:1000, NeuroMab, catalog number 75-146); IgG2b mouse anti-Ankyrin-G (1:300, NeuroMab, catalog number 75-147); chicken anti-βIV-spectrin (1:500, gift from Dr. M. Komada, Tokyo Institute of Technology, Tokyo, Japan); rabbit anti-parvalbumin (1:1000, Abcam catalog number Ab11427); guinea pig anti-NeuN (1:250, Synaptic System, catalog number 266 004); rabbit anti-calbindin (1:10000, Swant, catalog number CB38); mouse anti-calretinin (1:3000, Swant, catalog number 6B3); rabbit anti-PanNav (1:300, Alomone Labs, catalog number ASC-003); mouse anti-PanNav clone K58/35 (1:300, Sigma-Aldrich, catalog number S8809); rabbit anti-PanNav (1:300, Sigma-Aldrich, catalog number S6936); rabbit anti-Nav1.1 (1:500, Alomone Labs, catalog number ASC-001); mouse anti-Nav1.1 (1:500, NeuroMab, catalog number 75-023); rabbit anti-Nav1.2 (1:300, Alomone Labs, catalog number ASC-002); mouse anti-Nav1.2 (1:300, NeuroMab, catalog number 75-024); rabbit anti-Nav1.6 (1:300, Alomone Labs, catalog number ASC-009); mouse anti-Nav1.6 (1:300, NeuroMab, catalog number 75-026); mouse anti-Caspr (Neurexin IV) (1:500, NeuroMab, catalog number 75-001); mouse anti-MAP2 (1:500, Novus Biologicals, catalog number NBP2-25156); chicken anti-MAP2 (1:500, Synaptic System, catalog number 188 006); rabbit anti-Sox2 (1:1200, Millipore, catalog number AB5603); goat anti-DCX (1:400, Santa Cruz Biotechnology, catalog number sc-8066); rat anti-BrdU (1:1000, Abcam, catalog number ab6326). Day 2: Sections were washed five times with 1X PBS, then incubated with the appropriate secondary antibodies (1:250, Vector Laboratories) for 1 h in a 1X PBS solution containing 3% BSA and 0.1% Tween-20. The following isotype-specific secondary antibodies were used: Alexa 488-conjugated goat-anti-mouse IgG2a (for mouse Nav1.2), Alexa 568-conjugated goat-anti-mouse IgG1 (for mouse FGF14), Alexa 568-conjugated goat-anti-mouse IgG1 (for mouse FGF14) and Alexa 647-conjugated goat-anti-mouse IgG2a or Alexa 488-conjugated goat-anti-mouse IgG2b (for mouse Ankyrin-G NeuroMab, catalog number 75-146 and 75-147, respectively). After secondary antibody incubation, tissues were washed five more times with 1X PBS or TBS. Before mounting on Fisherbrand® Superfrost Plus glass microscope slides (Fisher Scientific), free-floating slices were rinsed with water and counterstained using the nuclear marker Topro-3 (1-3000, Life Technologies, Carlsbad, CA). Finally, glass slides were kept in an oven at 30–32°C for 10–15 min to dry and covered using Fisherfinest® Premium Cover Glass (Fisher Scientific) with ProLong® Gold anti-fade or ProLong® Gold anti-fade mountant with Dapi (Life Technologies, catalog number P36941). For Bromodeoxyuridine (BrdU) labeling, sections were incubated for 7 min in cold acetone then treated with 1 N HCL for 10 min, followed by 2 N HCl for 10 min at room temperature then 20 min at 37°C. Then, slices were incubated with borate for pH correction: 0.1 M borate buffer pH 8.5 for 10 min at room temperature, followed by the glass slide-mounted section immunolabeling procedure.

### Data reproducibility

For each protocol staining was first performed on tissue derived from one *Fgf14*^+∕+^ mouse and one *Fgf14*^−∕−^ as a control replicated on at least three separate brain sections. Then, once a protocol was elected as optimal for a given staining, experiments were repeated and confirmed on a higher number of animals (see legend of Tables 1–3).

### Confocal microscopy

Confocal images were acquired using a Zeiss LSM-510 META confocal microscope with a Fluar (5x/0.25) objective, a Plan-Apochromat (20x/0.75na) objective, a C-Apochromat (40x/1.2 W Corr) objective, and Plan-Apochromat (63x/1.46 Oil) objective. Multitrack acquisition was performed with excitation lines at 488 nm for Alexa 488, 543 nm for Alexa 568, and 633 nm for A647. Z-series stack confocal images were taken at fixed intervals: 0.6 μm for 40x, and 0.4 μm for 63x with the same pinhole setting for all three channels; frame size was either 1024 × 1024 or 512 × 512 pixels. All confocal images were processed using ImageJ US NIH (http://imagej.nih.gov/ij).

## Results

**Figure 1:**
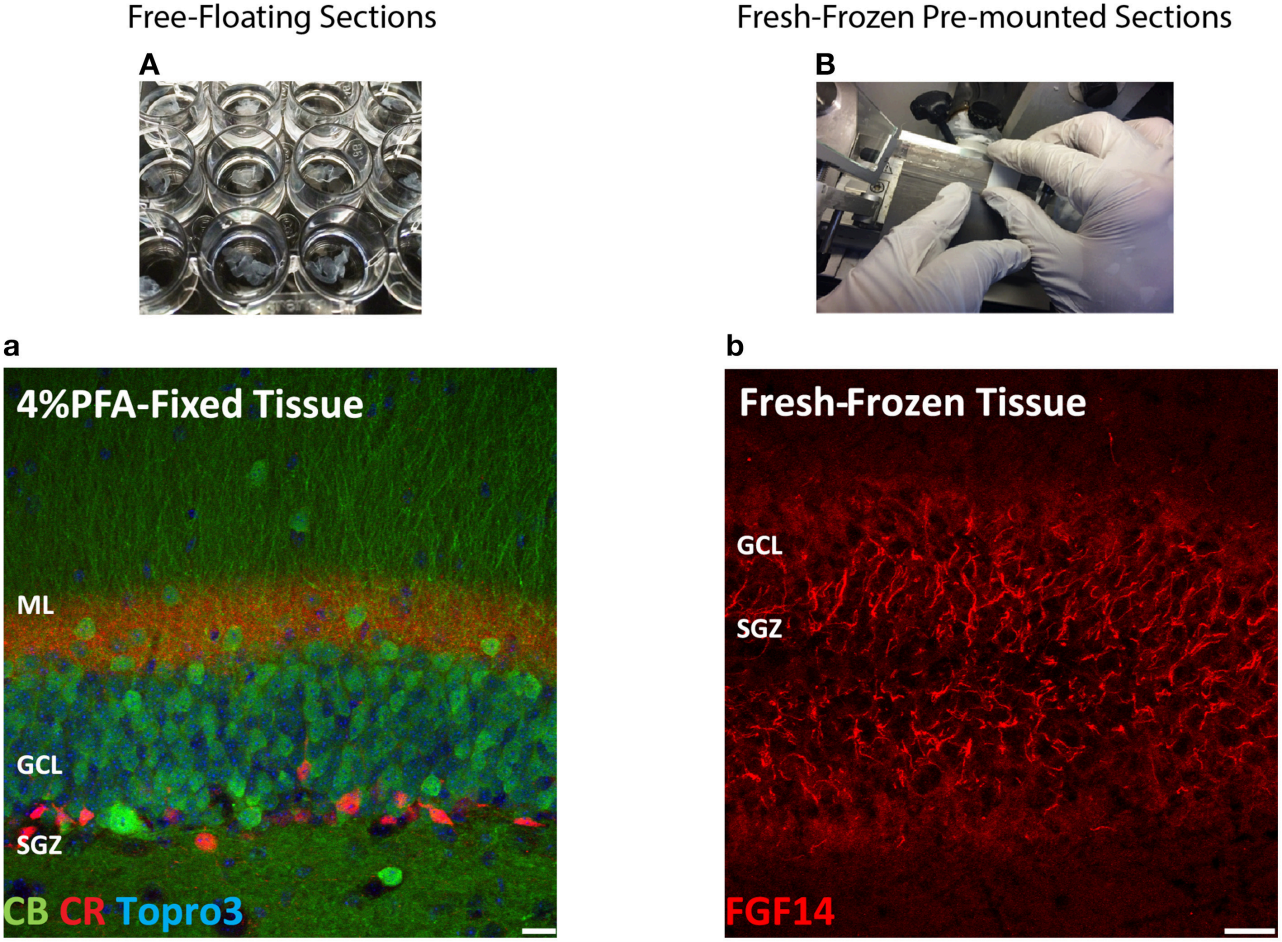
**Scheme and examples of routinely used brain tissue preparations for immunohistochemistry**. **(A)** Free-floating sections of 4% PFA perfusion-fixed mouse brain tissue are depicted in individual wells in a 24-well plate before mounting. **(a)** Representative confocal image of 4% PFA perfusion-fixed sections of the hippocampal DG immunolabeled with a rabbit anti-calbindin (CB) antibody visualized with an Alexa 488-conjugated secondary antibody (green), a mouse monoclonal anti-calretinin (CR) antibody visualized with an Alexa 568-conjugated secondary antibody (red) and Topro-3 nuclei staining (blue). **(B)** Fresh-frozen mouse brain slices directly adhered to positively charged glass slides. **(b)** Representative confocal image of a fresh-frozen section of the hippocampal DG, immunolabeled with a mouse monoclonal anti-FGF14 antibody visualized with an Alexa 568-conjugated secondary antibody. PFA, paraformaldehyde; DG, dentate gyrus; ML, molecular layer; GCL, granule cell layer; SGZ, sub-granular zone. Scale bars represent 20 μm.

**Scheme 1:**
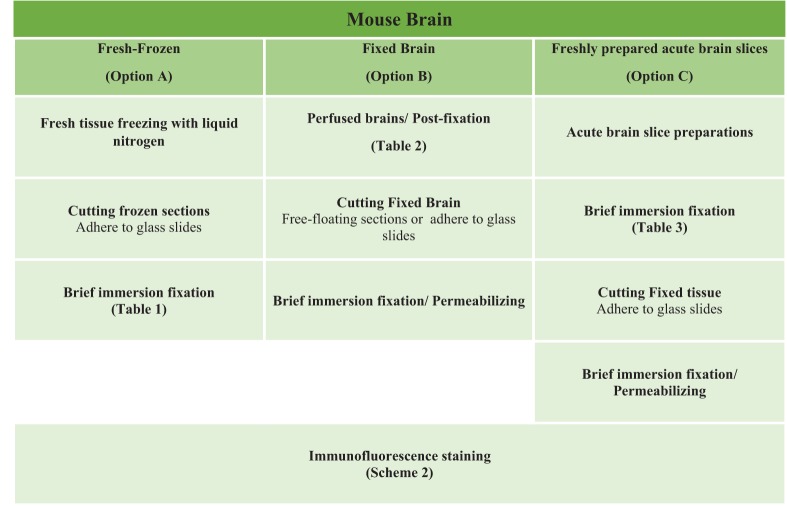
**Study design of the three options experimental procedures**. Mouse brain tissues were processed following three general procedures for fixation including **Option A** (fresh-frozen), **Option B** (perfusion-fixed), and **Option C** (freshly prepared acute brain slices) with up to ten variations in the type of fixative used for perfusion through the vascular system and/or for post-fixation treatment (Tables 1–3).

**Scheme 2:**
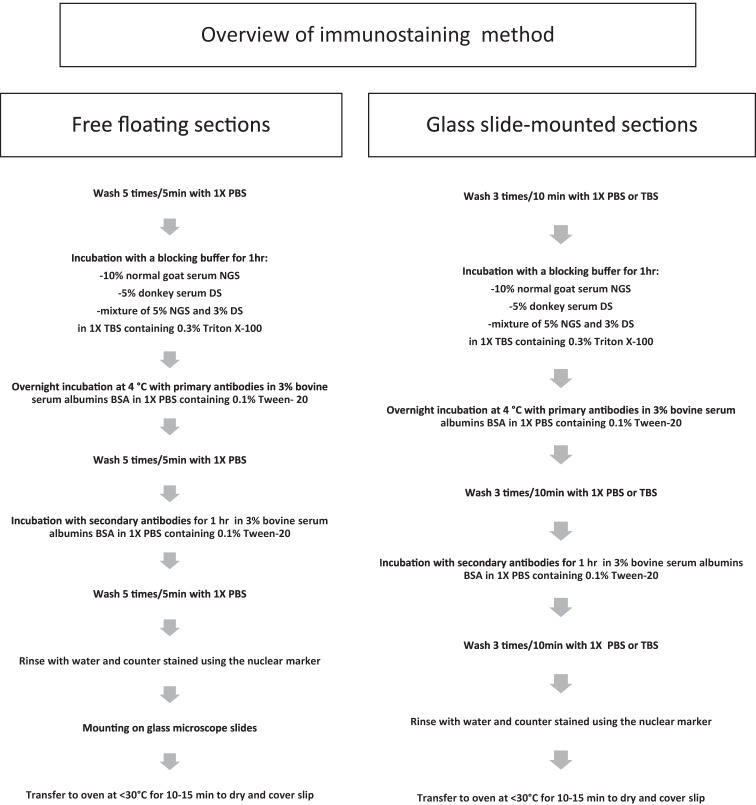
**Immunostaining procedure**. Workflow of the general immunostaining procedure used for free floating and glass slide pre-mounted brain sections.

The most routinely used fixed tissue preparations for immunostaining are 4% paraformaldehyde (PFA)-fixed free floating sections (Figures 1A,a) and fresh-frozen pre-mounted tissue slices that are captured during sectioning to adhere gently to positively charged glass slides (Figures 1B,b). The first method, which requires PFA perfusion of animals through the vasculature system, provides the highest quality fixation in terms of cellular and tissue preservation (Stradleigh and Ishida, 2015). Figure 1a illustrates a confocal image of triple immunolabeling of calbindin and calretinin-positive neurons along with Topro-3 nuclei staining in the mouse dentate gyrus. In this example, there is excellent preservation of the anatomical sub-layers and individual cell cytoarchitecture with the integrity of the cell soma and neurites; the thin filament-like structures that occupy the molecular layer and are visible in the green channel. However, this technique can be problematic for AIS staining where antigens are believed to be masked or damaged by fixation (Mojumder et al., 2007; Tian et al., 2014). Figure 1b illustrates an example of the distribution of the AIS protein FGF14 in the dentate gyrus whose immunolabeling was previously shown to require freshly frozen tissue fixation to warrant signal detection (Shavkunov et al., 2013; Xiao et al., 2013; Bosch et al., 2015). However, compared to PFA-fixed tissue, the dentate gyrus tissue preservation in freshly frozen tissue (Figure 1b) was far more limited (Figure 1a). To find an experimental condition that could combine advantages from both preparations, we designed a study consisting of three general classes of tissue preparations (Scheme 1): (i) fresh-frozen (option A); (ii) perfusion-based fixation (option B); (iii) freshly prepared acute brain slices (option C). Within these three general procedures more than 10 experimental conditions were tested (Tables 1–3). The overall immunostaining protocol is illustrated in (Scheme 2). As a control for the FGF14 staining, we used tissue derived from *Fgf14*^−∕−^ mice (see Supplementary Figures). We verified some of the other tested proteins using two different antibodies (Table 4).

**Table 4:** **Summary table of antigens, antibodies, and corresponding immunolabeling detection performance based on fixation procedures**.

**Comparing distribution patterns of FGF14 and selected AIS proteins and neuronal markers using different options of tissue fixation**				
**Target protein**	**Antibody: host, dilution and source**	**Fresh-frozen Option A**	**Perfusion-fixed Option B**	**Freshly prepared acute brain slices Option C**
		**Protein immunoreactivity was highly detected using**		
FGF14	Mouse anti-FGF14 (1:300, NeuroMabs, catalog number 75-096)	^*^ and ^**^ Prominent staining in AIS (Figures 1B, 2C–F; Supplementary Figure 1A)	^++^, ^++++^, and ^±^ Prominent staining in AIS and soma (Figures 3B,D, 4–8; Supplementary Figures 2–6)	^#^ Prominent staining in AIS and soma (Figures 9A–C)
Ankyrin-G	Mouse anti-Ankyrin-G (1:1000, NeuroMabs, catalog number 75-146)	Not tested	^++^, ^+++^, and ^±^ Prominent staining in AIS (Figures 6, 7C; Supplementary Figures 3, 5C, 6C,D)	^#^ Prominent staining in AIS (Figures 9A–D)
Ankyrin-G	Mouse anti-Ankyrin-G (1:300, NeuroMabs, catalog number 75-147)	^*^ Prominent staining in AIS ^‡^	^++^ and ^+++^ Prominent staining in AIS	^#^ Prominent staining in AIS
β-IV-spectrin	Chicken anti-β-IV-spectrin (1:500, gift from Dr. M. Komada, Tokyo Institute of Technology, Tokyo, Japan)	^*^ Prominent staining in AIS ^‡^ (Figure 2D)	^++^, ^+++^, ^++++^, and ^±^ Prominent staining in AIS (Supplementary Figures 6A,B)	Not tested
Parvalbumin	Rabbit anti-Parvalbumin (1:1000, Abcam catalog number Ab11427)	^**^ weak staining in the soma (Figure 2F)	^++^ and ^++++^ Prominent staining of somata, dendrites, and AIS; ^+++^ Prominent staining of somata and dendrites (Figures 3H–I, 4)	Not tested
NeuN	Guinea pig anti-NeuN (1:250, Synaptic System, catalog number 266 004)	Not tested	^++^, ^+++^, and ^±^ Prominent staining of somata (Figures 3G, 6, 7C; Supplementary Figures 3, 6A)	^#^ Prominent staining of somata (Figure 9D)
Calbindin	Rabbit anti-calbindin (1:10,000, Swant, catalog number CB38)	Not detectable	^++^, ^+++^, ^++++^, and ^+++++^ Prominent staining of somata and dendrites (Figures 1A, 3F,J, 5; Supplementary Figures 5A,B)	Not tested
Calretinin	Mouse anti-calretinin (1:3000, Swant, catalog number 6B3)	Not tested	^+++^ Prominent staining of somata and dendrites (Figure 1A)	Not tested
Sox2	Rabbit anti-Sox2 (1:1200, Millipore, catalog number AB5603)	Not tested	^++^ and ^+++^ Prominent staining of somata (Figure 7A)	Not tested
DCX	Goat anti-DCX (1:400, Santa Cruz Biotechnology, catalog number sc-8066)	Not detectable	^++^ and ^+++^ Prominent staining of somata and dendrites (Figure 7B)	Not tested
BrdU	Rat anti-BrdU (1:1000, Abcam, catalog number ab6326)	Not tested	^++^ and ^+++^ with (DNA denaturation protocol) Prominent staining of somata (Figure 7C)	Not tested
PanNav	Rabbit anti-PanNav (1:300, Alomone Labs, catalog number ASC-003)	^*^ Prominent staining in soma and AIS ^‡^ (Supplementary Figure 1A)	^++^ Prominent staining of somata, and weak detection in the AIS ^‡^ (Supplementary Figure 4A)	^#^ Prominent staining in the soma
PanNav	Rabbit anti-PanNav (1:300, Sigma-Aldrich, catalog number S6936)	^*^ (Wildburger et al., 2015; Shavkunov et al., 2013)	^++^ Prominent staining of somata, and weak detection in the AIS ^‡^ (Figure 8A)	^#^ Prominent staining in the soma
PanNav	Mouse anti-PanNav clone K58/35 (1:300, Sigma-Aldrich, catalog number S8809)	Not tested	^++^ Weak detection in the AIS; ^±^ Prominent staining in AIS (Supplementary Figures 6A,B, 7A,B)	Not tested
Nav1.1	Rabbit anti-Nav1.1 (1:500, Alomone Labs, catalog number ASC-001)	^*^ Prominent in soma and weak in AIS ^‡^	^++^ Prominent staining of somata, and weak detection in the AIS (Figure 8B)	^#^ Prominent staining in the soma and weak in AIS
Nav1.1	Mouse anti-Nav1.1 (1:500, NeuroMab, catalog number 75-023)	Not tested	^++^ Prominent staining of somata, and weak detection in the AIS	Not tested
Nav1.2	Rabbit anti-Nav1.2 (1:300, Alomone Labs, catalog number ASC-002)	^*^ weak detection ^‡^	^++^ Prominent staining in AIS of the neurons in the cerebellum ^‡^ (Figure 8C; Supplementary Figure 4C)	Not tested
Nav1.2	Mouse anti-Nav1.2 (1:300, NeuroMab, catalog number 75-024)	^*^ Wildburger et al., 2015	^++^ weak detection ^‡^ (Supplementary Figure 4B)	^#^ weak detection
Nav1.6	Rabbit anti-Nav1.6 (1:300, Alomone Labs, catalog number ASC-009)	^*^ Prominent staining in AIS ^‡^	^++^ and ^±^ Prominent staining in AIS and Node of Ranvier (Figure 8D; Supplementary Figures 4D, 7A,B)	^#^ Prominent staining in AIS (Figures 9A–D)
Nav1.6	Mouse anti-Nav1.6 (1:300, NeuroMab, catalog number 75-026)	Not tested	^++^ Prominent staining in AIS ^‡^	^#^ Prominent staining in AIS
Caspr	Mouse anti-Caspr (1:500, NeuroMab, catalog number 75-001)	Not tested	^++^ Prominent staining of the Node of Ranvier ^‡^ (Supplementary Figure 4D)	Not tested
MAP2	Mouse anti-MAP2 (1:500, Novus Biologicals, catalog number NBP2-25156)	Not tested	^++^ Prominent staining of somata and dendrites ^‡^ (Supplementary Figure 5C)	Not tested
MAP2	Chicken anti-MAP2 (1:500, Synaptic System, catalog number 188 006)	Not tested	^++^ Prominent staining of somata and dendrites ^‡^ (Supplementary Figures 5A,B)	Not tested

Protein immunoreactivity was detected using the following fixation and post-fixation procedures:

* *Fresh-frozen sections immersed in acetone (7 min) (Shavkunov et al., 2013; Wildburger et al., 2015)*.

** *Fresh-frozen sections immersed in acetone (7 min) followed by methanol (7 min)*.

++ *Perfusion-fixed tissue: animal was perfused intracardially with commercially available 1% formaldehyde + %0.5 methanol (Master-Tech Scientific), then sections immersed in acetone (7 min)*.

+++ *Perfusion-fixed tissue: animal was perfused intracardially with 4% PFA*.

++++ *Perfusion-fixed tissue: animal was perfused intracardially with 4% PFA, and then sections immersed in acetone (7 min)*.

+++++ *Perfusion-fixed tissue: animal was perfused intracardially with commercially available Optimal Fix^TM^ (Master-Tech Scientific), and then sections immersed in acetone (7 min)*.

± *Brains were extracted then immersed in 4% PFA for 30 min followed by incubation in 20–30% sucrose overnight. Then sections were permeabilized with 1 % Triton X-100, 0.5 % Tween in 1X PBS for 7–10 min*.

# *Acute brain slices: animal was perfused intracardially with ACSF, and then slices immersed in commercially available 1% formaldehyde + 0.5 methanol (Master-Tech Scientific) (30 min), followed by overnight incubation in 20–30% sucrose, then sections immersed in acetone (7 min)*.

‡ *Only one condition tested*.

### Option A: optimization of FGF14 staining in fresh-frozen tissue

**Figure 2:**
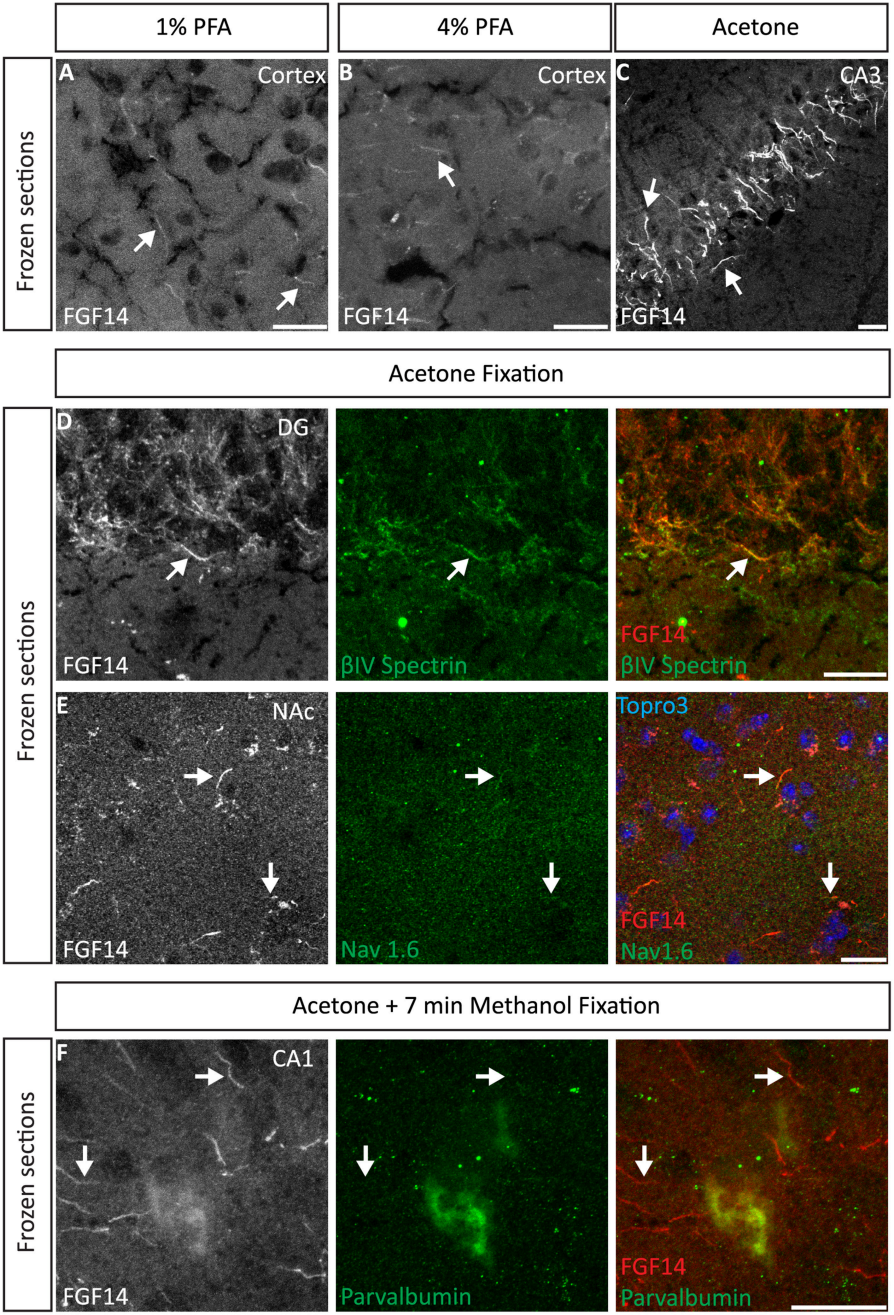
**Representative examples of double immunofluorescence staining of mouse brain fresh-frozen sections followed by the indicated post-fixative treatments. (A)** Weak detection of FGF14 immunoreactivity at the AIS (arrow) in cells in the mouse cortical region using 1% PFA post-fixed treatment (Scheme 1, Option A, first column of Table 1). **(B)** Weak detection of FGF14 immunoreactivity at the AIS (arrow) in cells of the mouse cortical region using 4% PFA post-fixed treatment (Scheme 1, Option A, second column of Table 1). **(C)** Optimal detection of FGF14 immunoreactivity in the CA3 hippocampal region following acetone-based brief post-fixation treatment (Scheme 1, Option A, third column of Table 1). **(D)** Representative confocal images of double immunostaining of the DG using acetone-based post-fixation treatment. The gray and red channels represent FGF14 immunoreactivity visualized with an Alexa 568-conjugated secondary antibody. The green channel represents βIV-spectrin immunoreactivity visualized with an Alexa 488-conjugated secondary antibody. Arrows show co-localization between FGF14 and βIV-spectrin at the AIS. Green and red channel overlay images are shown on the right. **(E)** Representative confocal images of double immunostaining of the NAc using acetone-based (without methanol) post-fixation treatment (Scheme 1, Option A, third column of Table 1). The gray and red channels represent FGF14 immunoreactivity, the green channel represents Nav1.6 (primary antibody from Alomone Labs) visualized (weakly) with an Alexa 488-conjugated secondary antibody. The blue represents Topro-3 nuclear staining shown in the green, red, and blue image overlay on the right. Arrows show co-localization between FGF14 and Nav1.6 at the AIS. **(F)** Representative confocal images of double immunostaining of a zoomed area of the CA1 hippocampal region using acetone + methanol-based post-fixation treatment (Scheme 1, Option A, fourth column of Table 1). The gray and red channels represent FGF14 immunoreactivity visualized with an Alexa 568-conjugated secondary antibody. The green channel represents parvalbumin immunoreactivity visualized with an Alexa 488-conjugated secondary antibody. Arrows show localization of FGF14 at the AIS in areas around the parvalbumin soma. Green and red channel overlay images are shown on the right. Note an FGF14 positive halo overlays with somatic parvalbumin staining suggesting localized co-expression of the two proteins in cytoplasmic regions. Arrows indicate FGF14, βIV-spectrin, and/or Nav1.6 signals at the axon initial segment (AIS). DG, dentate gyrus; NAc, nucleus accumbens; PFA, paraformaldehyde. Scale bars represent 20 μm.

We began our study exploring different post-fixative conditions following the fresh-frozen procedure (**Option A**). Mouse brains were dissected and rapidly dipped in liquid nitrogen, then tissue was sectioned using a cryostat set at 10–15 μm thick slices. Tissue slices were allowed to adhere to glass slides and exposed to additional fixatives/permeabilizing agents in five different combinations: (i) 1% PFA + acetone; (ii) 4% PFA + acetone; (iii) acetone alone; (iv) acetone followed by methanol; (v) 2% PFA + 0.2% glutaraldehyde followed by acetone treatment. A summary of these procedures can be found in Table 1. Out of the five conditions, the glutaraldehyde mixed with paraformaldehyde fixation failed to show any detectable FGF14 AIS immunoreactivity (data not shown). The 1% or 4% PFA treatment did not completely prevent FGF14 immunoreactivity at the AIS (Figures 2A,B), but was associated with a high fluorescence background, possibly from tissue auto-fluorescence. In contrast, the cold acetone alone treatment improved the FGF14 immunosignal significantly at the AIS in many brain regions including the CA3 hippocampus (Figure 2C).

Next, we conducted a series of double staining experiments using the latter condition, and succeeded in detecting other AIS proteins such as βIV-spectrin in the DG (Figure 2D), Nav1.6 in the nucleus accumbens (NAc; Figure 2E) and PanNav in the cortex (Supplementary Figure 1A). To further assess the validity of this procedure, we conducted double staining experiments using cell type-specific neuronal markers such as parvalbumin (a marker of a subclass of inhibitory cells that acts as a fast intracellular Ca^2+^ buffer), calbindin (a marker of a subclass of inhibitory cells that acts as a slow intracellular Ca^2+^ buffer; Chard et al., 1993) and doublecortin (a marker of immature neurons; Brown et al., 2003). Yet, we were unable to detect signals from any of these cell markers (data not shown). We then attempted to improve our detection method by adding a second post-fixation procedure by rapidly immersing acetone-fixed slices in cold methanol. As a result, we were able to detect a weak signal for parvalbumin in the soma (Figure 2F), but the overall quality of the staining was suboptimal. Consistent with previous studies from our laboratory (Shavkunov et al., 2013), our results from fresh-frozen preparations in five different fixation treatments suggest that cold acetone fixation with or without a subsequent light fixation in cold methanol are ideal conditions for AIS studies, but are sub-optimal for studying other cell type markers.

### Option B: optimization of FGF14 staining fixed tissue

**Figure 3:**
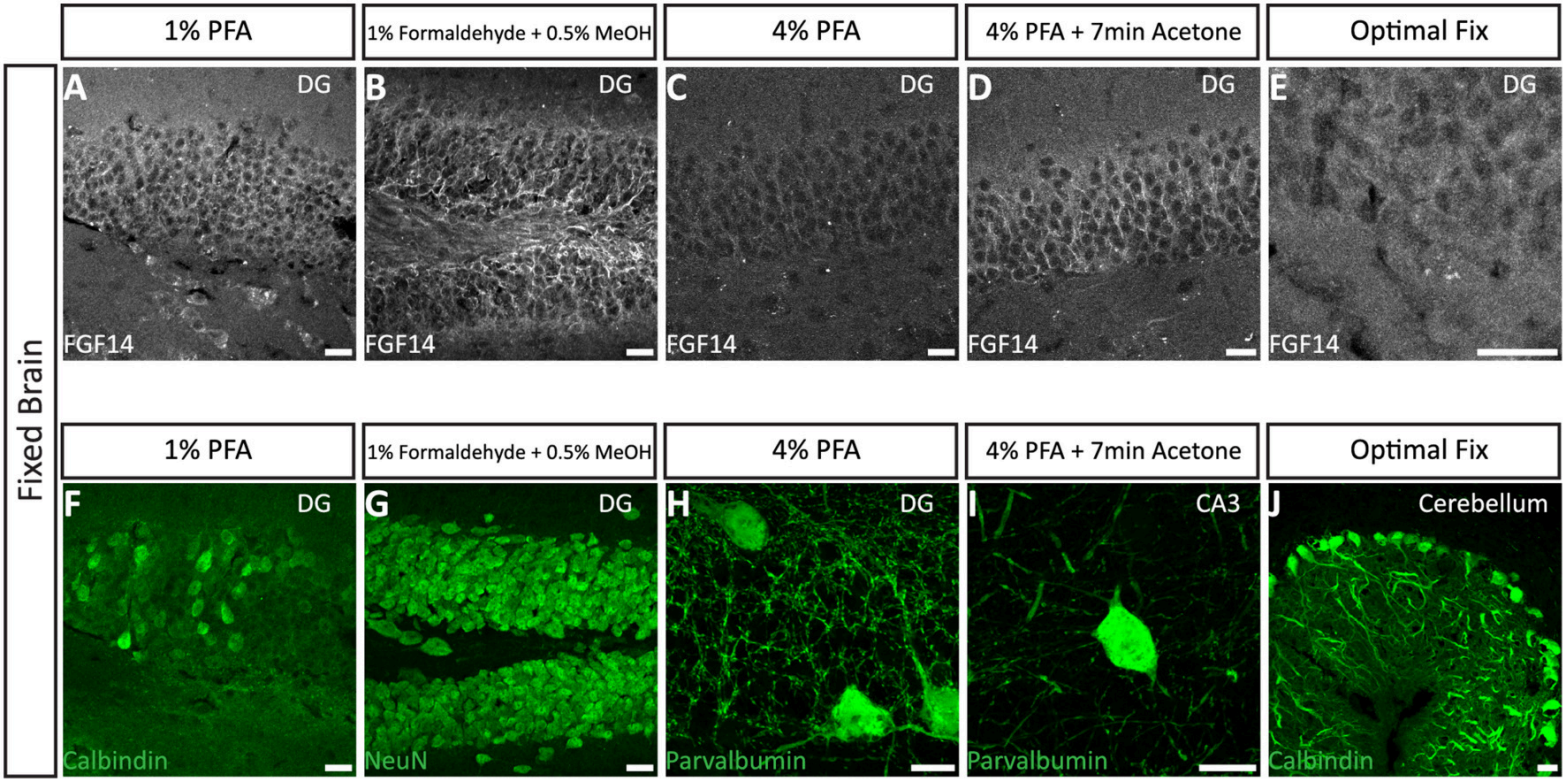
**Representative examples of immunofluorescence staining of mouse fixed brain sections followed by indicated post-fixation treatments. (A)** 1% PFA perfused-brain fixation revealed weak detection of FGF14 at the AIS in the DG region (Scheme 1, Option B, first column of Tale 2). **(B)** Light fixation with a mixture containing 1% formaldehyde and 0.5% methanol (diluted from commercially available mixture of 37% formaldehyde in PBS, pH = 7.4) resulted in robust staining of FGF14 in the DG region (Scheme 1, Option B, second column of Table 2). **(C)** FGF14 immunoreactivity is almost non-detectable in the DG upon 4% PFA perfused-fixed sections [Scheme 1, Option B, third column of Table 2 (left)]. **(D)** FGF14 immunoreactivity is enhanced in 4% PFA perfused-brain followed by brief acetone post-fixation treatment [Scheme 1, Option B, third column of Table 2 (right)]. **(E)** FGF14 immunoreactivity is non-detectable in Optimal Fix™ perfusion conditions (Scheme 1, Option B, fifth column of Table 2). **(F)** The green channel represents calbindin immunoreactivity visualized with an Alexa 488-conjugated secondary antibody in the DG. **(G)** Enhanced NeuN staining visualized with an Alexa 488-conjugated secondary antibody in 1% formaldehyde with 0.5% methanol fixation in the DG. **(H)** Detection of parvalbumin immunoreactivity visualized with an Alexa 488-conjugated secondary antibody in the soma and dendrites with 4% PFA fixed sections in the DG. **(I)** Parvalbumin immunoreactivity in the soma and dendrites in the CA3 hippocampal region using 4% PFA followed by brief acetone post-fixation treatment. **(J)** Calbindin immunoreactivity in the cerebellum using Optimal Fix™ perfusion. DG, dentate gyrus; PFA, paraformaldehyde. Scale bars represent 20 μm.

We next tested the immunostaining of FGF14 in samples derived from tissue perfused for a short period of time (10 min) via the vascular system of the animal using different fixatives followed by post-fixation/permeabilization treatments (**Option B**). The variables that defined these different treatments included the type of material, its concentration and/or the treatment time (Table 2). These protocols were inspired by trial-and-error attempts of our own laboratory experience or published data on FGF14 (Shakkottai et al., 2009; Xiao et al., 2013; Bosch et al., 2014, 2015). In the three conditions where the perfused fixative concentration was weak (1% PFA, commercially available 1% formaldehyde + 0.5% methanol, and commercially available Optimal Fix™) we faced tissue-tearing issues and had to use the adhered instead of free-floating tissue approach. We posited that perfusing animals with a low concentration of PFA might not be sufficient to maintain thin tissue sections intact in the ethylene glycol-based cryoprotectant solution. In the first condition, consisting of 1% PFA perfusion, the FGF14 immunostaining at the AIS was weak, but detectable (Figure 3A), the tissue integrity was not ideal, and the neuronal cell type marker detection (calbindin) was suboptimal (Figure 3F vs. Figure 1a). The second condition consisting of the 1% formaldehyde + 0.5% MeOH (MasterTech Scientific) fixation showed a highly distinct FGF14 signal at the AIS (Figure 3B) with overall well-preserved tissue morphology and good neuronal marker detection (Figure 3G). On the contrary, the routinely used 4% PFA fixation yielded almost non-detectable FGF14 AIS signals (Figure 3C), but provided great tissue preservation and cell type marker counterstaining (Figure 3H). Interestingly, incubating 4% PFA perfused tissue sections with cold acetone for <10 min enhanced the detection of FGF14 signal at the AIS without compromising tissue structure (Figure 3D). In the same preparation, parvalbumin immunoreactivity in both soma and dendrites was well-defined (Figure 3I). In the last condition, animals were perfused with nontoxic alcohol-based Optimal Fix™ solution (MasterTech Scientific). This method was not effective in revealing FGF14 staining and did not preserve the tissue, but the resulting calbindin staining was good (Figure 3J). From these experiments, we can conclude that 4% PFA (24 h post-fixation in 4% PFA) + cold acetone and 1% formaldehyde + 0.5% MeOH (1 h post-fixation in same fixative) were two experimental conditions that provided high detectable FGF14 signal at the AIS, and great preservation of tissue integrity. We then conducted additional double labeling experiments in various brain regions using either 4% PFA + cold acetone or 1% formaldehyde + 0.5% MeOH.

#### FGF14 double staining using the 4% PFA perfusion + post-fix cold acetone condition

**Figure 4:**
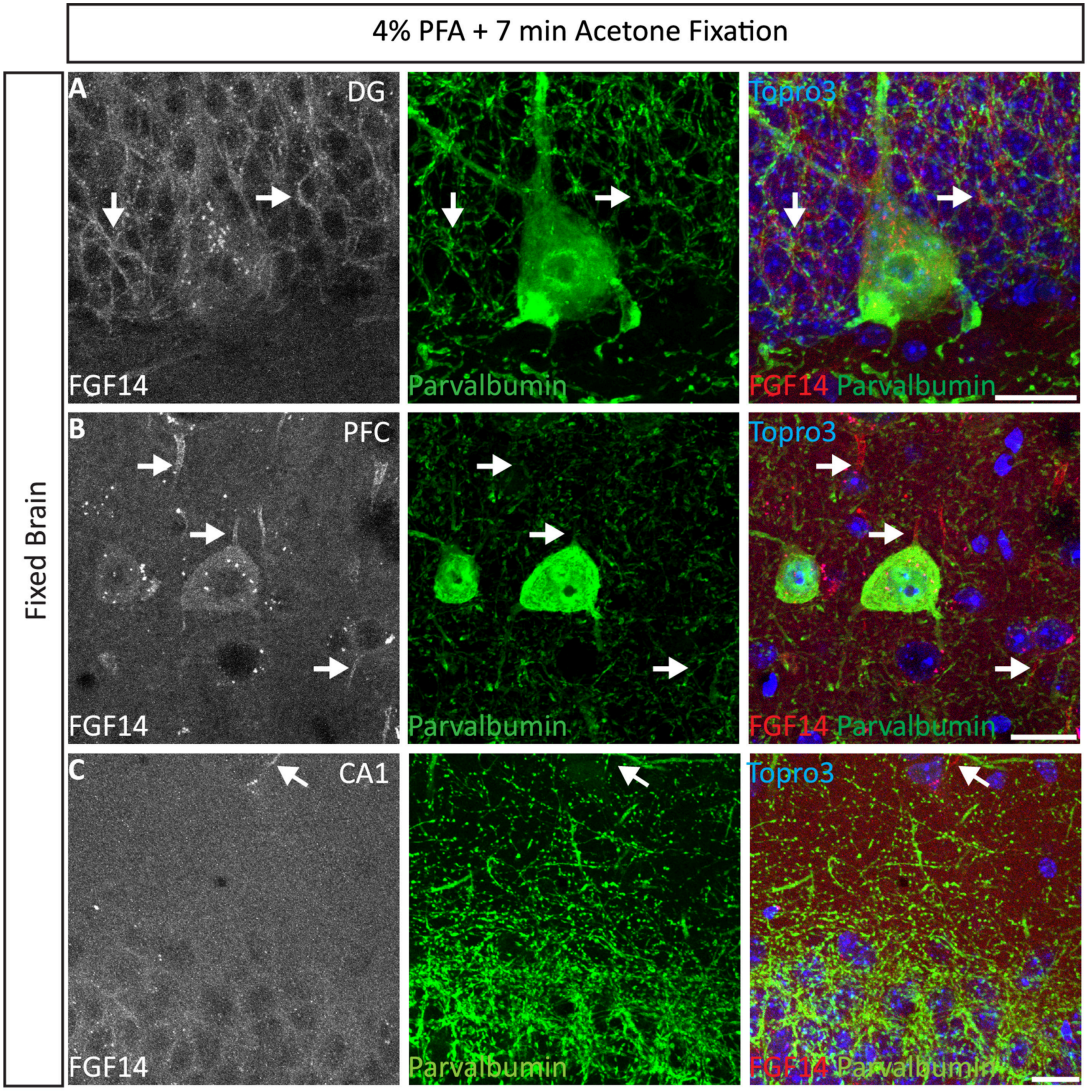
**Representative examples of double immunofluorescence staining of mouse brain tissue using 4% PFA perfusion and acetone-based post-fixation treatment**. For the entire figure, the gray and red channels represent FGF14 immunoreactivity visualized with an Alexa 568-conjugated secondary antibody; the green channel represents parvalbumin immunoreactivity visualized with an Alexa 488-conjugated secondary antibody; image overlaid of the green, red, and blue channel (representing Topro-3 nuclear staining) in the DG region **(A)**, PFC region **(B)**, and CA1 region **(C). (C)** The same staining and immunolabelling used in **(A,B)** reveals a rather weak signal corresponding to FGF14 immunoreactivity, but selective parvalbumin labeling of somata and dendrites (green) in the CA1 hippocampal region. Arrows show FGF14 signals at the axon initial segment (AIS). DG, dentate gyrus; PFC, pre-frontal cortex; PFA, paraformaldehyde. Scale bars represent 20 μm.

Interestingly, the 4% PFA + cold acetone condition did not elicit the same results in all brain regions. For instance, it provided higher quality double staining (FGF14 + parvalbumin) in the DG region (Figure 4A) and prefrontal cortex (PFC, Figure 4B) than in the CA1 hippocampal region (Figure 4C). Furthermore, 4% PFA + cold acetone showed good detection of FGF14 and calbindin in the prefrontal cortex (PFC, Supplementary Figure 5A, upper set), but not in the CA1 hippocampal region (Supplementary Figure 5A, lower set). This discrepancy may be due to the anatomy of the CA1 hippocampal region, where laminations are tight and denser compared to other brain regions (Rho et al., 2010), rather than variability in FGF14 expression (Shavkunov et al., 2013).

#### FGF14 double and triple staining using 1% formaldehyde + 0.5% MeOH perfusion condition

**Figure 5:**
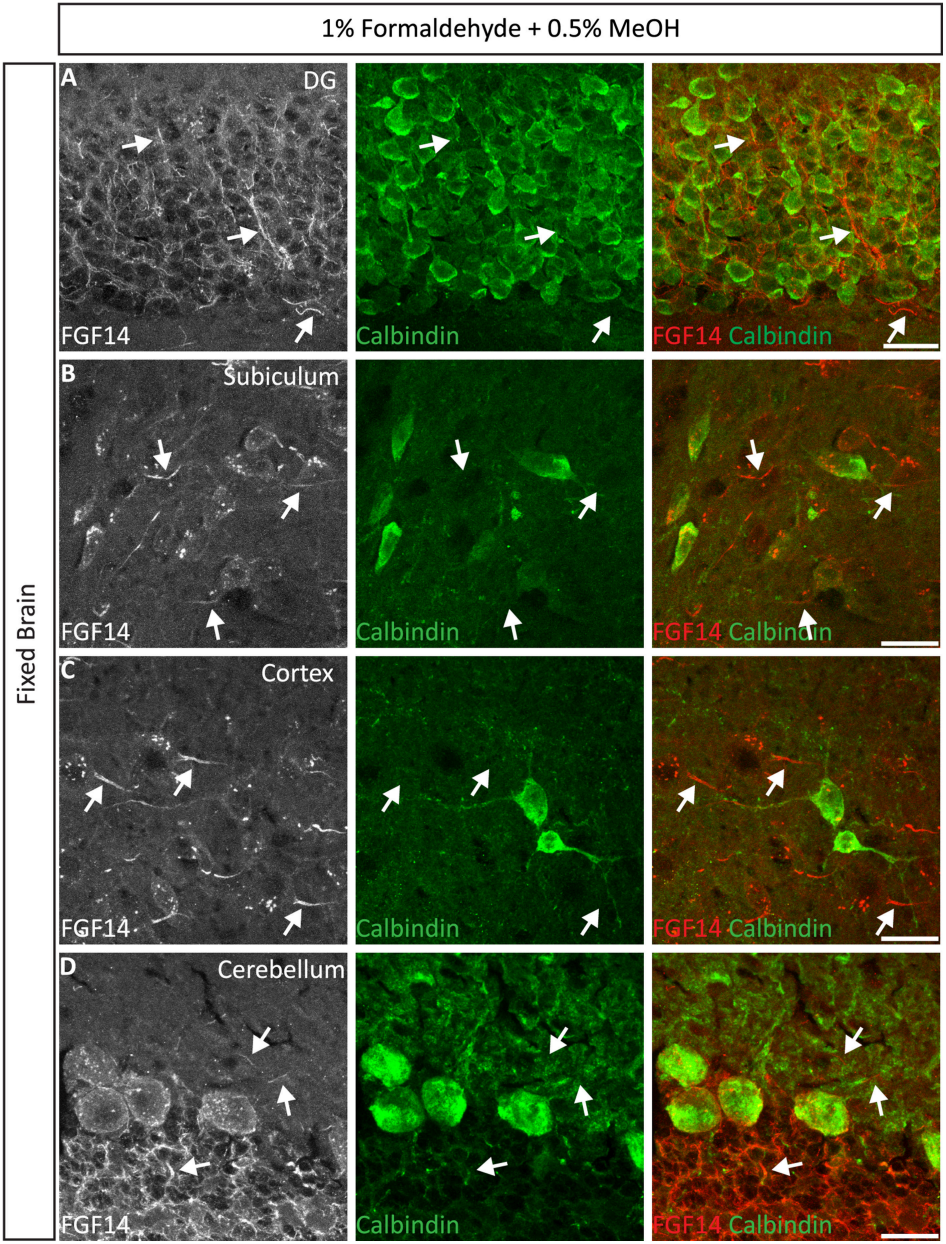
**Representative examples of double immunofluorescence staining of mouse brain tissue using 1% formaldehyde and 0.5% MeOH fixation. (A–D)** For the entire figure, the gray and red channels represent FGF14 immunoreactivity and the green calbindin in indicated brain regions. The corresponding green and red merged images are shown in the right column. Arrows indicate FGF14 signal at the axon initial segment (AIS). DG, dentate gyrus. Scale bars represent 20 μm.

**Figure 6:**
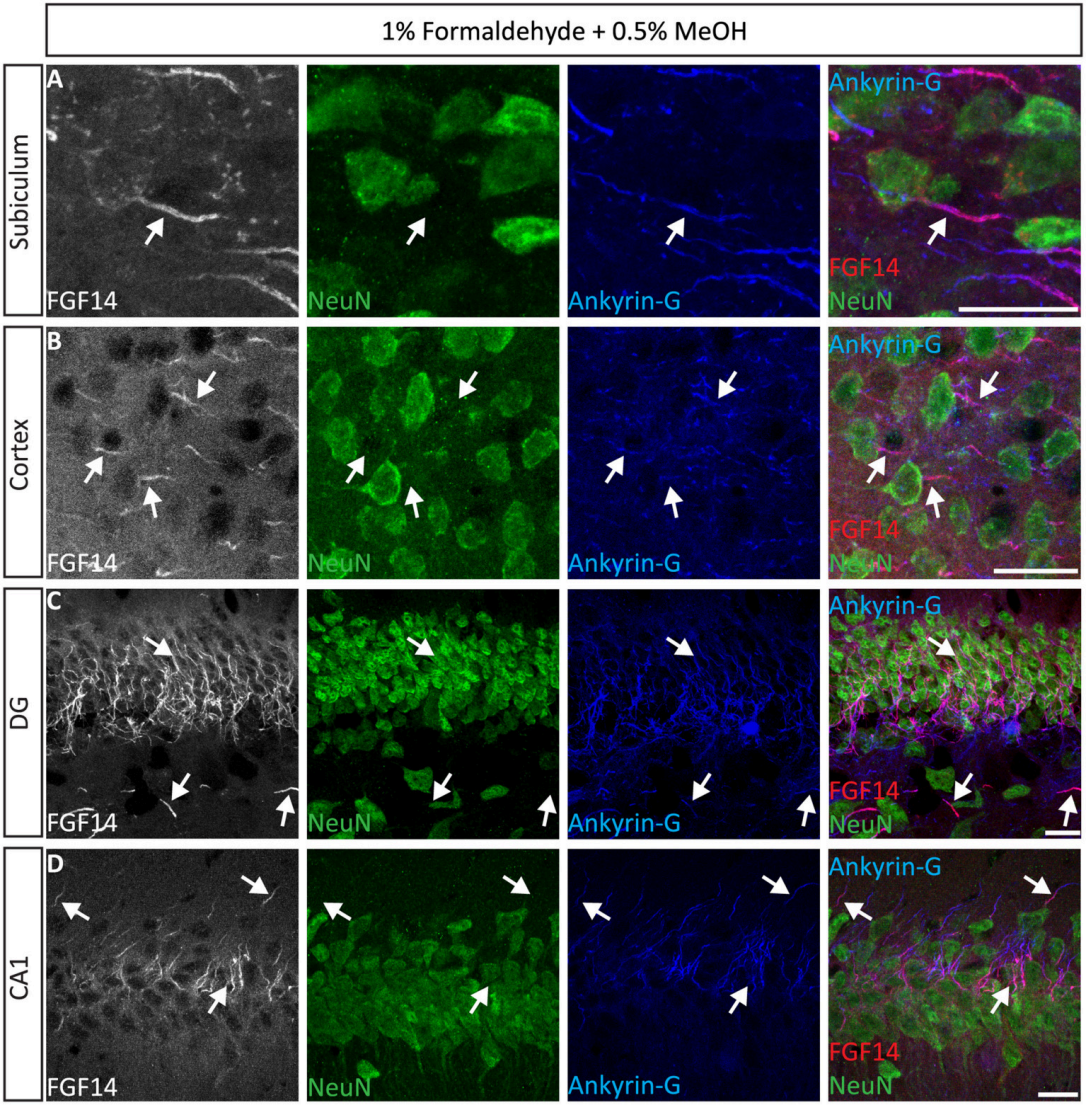
**Representative examples of triple immunofluorescence staining of mouse brain tissue using 1% formaldehyde and 0.5% MeOH fixation. (A–D)** For the entire figure, the gray and red channels represent FGF14 immunoreactivity, the green NeuN, and blue Ankyrin-G (NeuroMab, catalog number 75-146) in indicated brain regions. The corresponding multichannel overlaid images are shown in the right column. DG, dentate gyrus; NeuN, Neuronal marker. Scale bars represent 20 μm.

**Figure 7:**
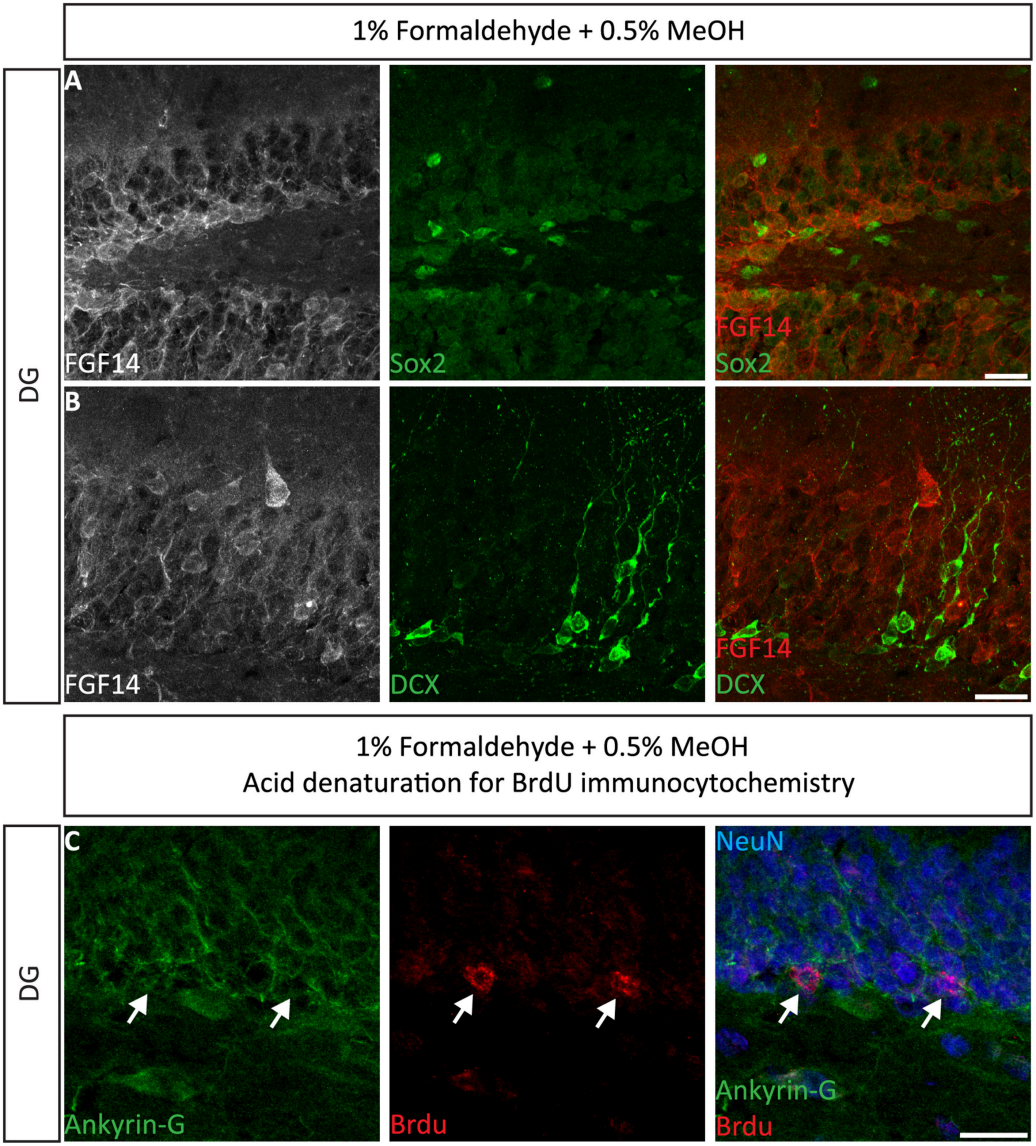
**Immunolabeling of FGF14 and selected neurogenesis markers in the DG using 1% formaldehyde and 0.5% MeOH fixation**. **(A)** The gray and red channels represent FGF14 immunoreactivity visualized with an Alexa 568-conjugated secondary antibody, and the green channel represents Sox2, or DCX in **(B)**, visualized with an Alexa 488-conjugated secondary antibody. Green and red channels overlaid images are shown in the right column of both **(A,B)**. **(C)** The green channel represents Ankyrin-G (NeuroMab, catalog number 75-146) visualized with an Alexa 488-conjugated secondary antibody. The red channel represents BrdU immunoreactivity visualized with an Alexa 568-conjugated secondary antibody and the blue represents NeuN visualized with an Alexa 647-conjugated secondary antibody. DG, dentate gyrus; Sox2, Sex determining region Y-Box 2; DCX, doublecortin; BrdU, Bromodeoxyuridine; NeuN, Neuronal marker. Scale bars represent 20 μm.

Next, we extended the evaluation of our second successful protocol consisting of intracardial perfusion with a commercially available solution containing 1% formaldehyde + 0.5% MeOH, then sections were immersed in lightly-fixative of cold acetone before immunostaining. As shown in Figure 5, this condition provided accurate detection of FGF14 in the soma and at the AIS along with calbindin immunolabeling across the DG (Figure 5A), subiculum (Figure 5B), cortex (Figure 5C), and cerebellum (Figure 5D). With the same method in triple staining experiments we successfully detected FGF14, Ankyrin-G and NeuN immunoreactivity in the subiculum (Figure 6A), cortex (Figure 6B), DG (Figure 6C) and the CA1 hippocampal region (Figure 6D). We further analyzed the detection capacity of the 1% formaldehyde + 0.5% MeOH method using Microtubule-associated protein 2 (MAP2), which labels the soma and dendrites, in combination with calbindin and the AIS marker Ankyrin-G. As shown in Supplementary Figures 5B,C, FGF14 immunosignals were sharply detected, along with MAP2, in well-defined calbindin-positive cells in the DG and the cortex. Using the same procedure, we also extended our evaluation of FGF14 staining in conjunction with cell markers of neurogenesis, some of which are notoriously hard to work with. As a result, we were able to detect high-quality signals for both FGF14 and Sex Determining Region Y-Box 2 (Sox2), a marker of early neuronal progenitors (Figure 7A), and for doublecortin (DCX), a marker of immature neurons (Figure 7B). Consistently with another study conducted in our laboratory, we found that the expression pattern of FGF14 varies across different stages of adult neurogenesis (Alshammari et al., 2015). We also attempted the same protocol for detecting FGF14 in BrdU^+^ dividing stem cells but failed to detect any signal (from FGF14 immunolabeling). That could be due to lack of FGF14 expression in dividing cells (although this seems unlikely since FGF14 is expressed in Sox2 early progenitors, Figure 7A) or to interference of the acidic treatment required for DNA denaturation and BrdU detection with the FGF14 epitope. However, we succeeded in detecting another AIS component, Ankyrin-G, in BrdU-positive cells (Figure 7C).

**Figure 8:**
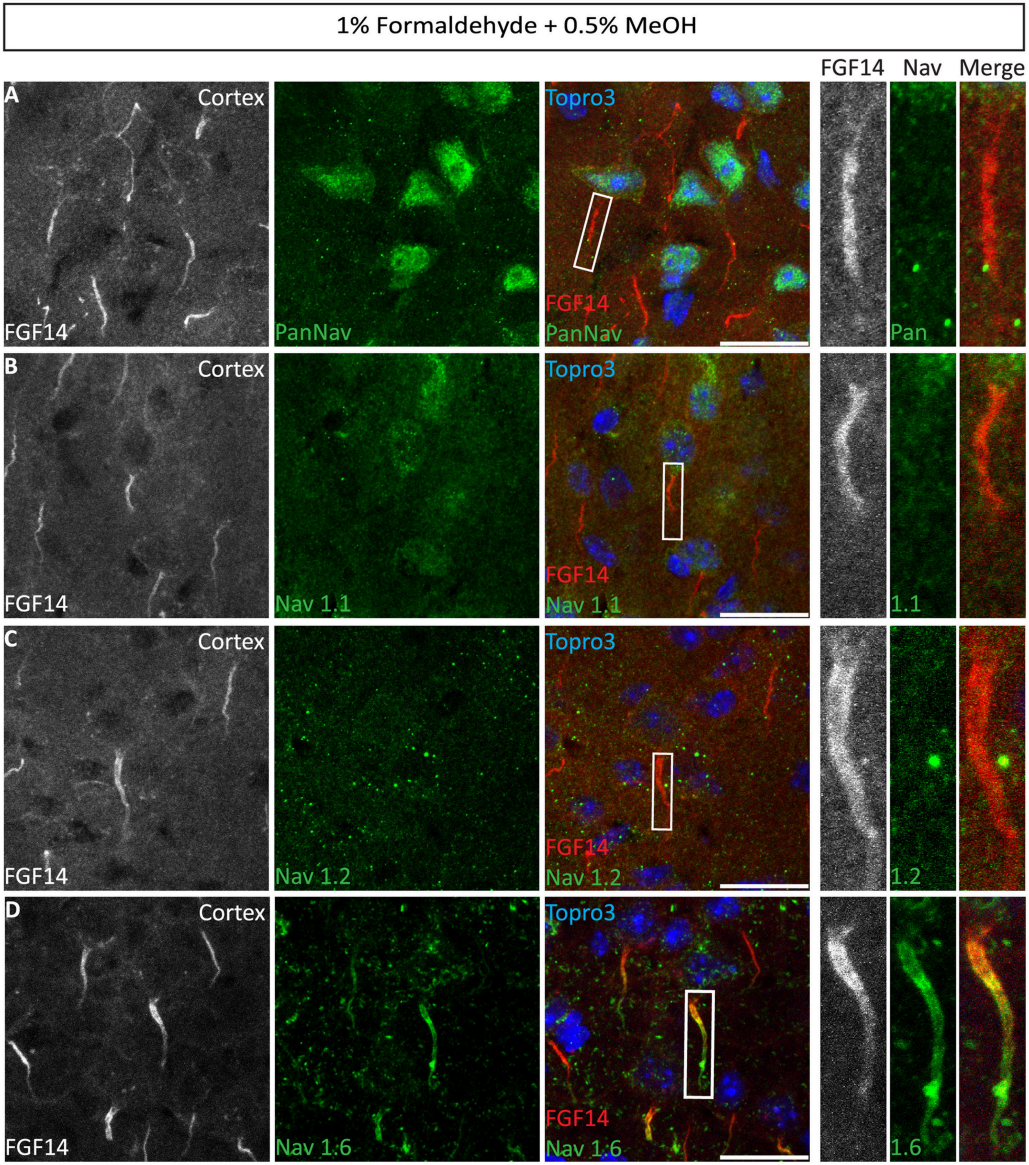
**Co-localization of FGF14 and Nav1.6 in mouse cortex using 1% formaldehyde and 0.5% MeOH fixation**. **(A–D)** The gray and red channels represent FGF14 immunoreactivity visualized with an Alexa 568-conjugated secondary antibody, the green channel represents PanNav (Sigma-Aldrich, rabbit anti PanNav, catalog number S6936) in **(A)**, Nav1.1 (Alomone Labs) in **(B)**, Nav1.2 (Alomone Labs) in **(C)**, and Nav1.6 (Alomone Labs) in **(D)** visualized with an Alexa 488-conjugated secondary antibody and the blue represents Topro3 nuclear staining in the cortex. Right panels represent overlaid images (third column from the left) and high magnification of boxed ROI from the merged images. Scale bars represent 20 μm.

Numerous studies indicate a prominent role of FGF14 in regulating Nav channel targeting and function in a Nav isoform-specific manner (Laezza et al., 2007, 2009; Diwakar et al., 2009; Shavkunov et al., 2013; Hsu et al., 2014; Bosch et al., 2015). Thus, we evaluated our 1% formaldehyde + 0.5% MeOH protocol for the double labeling of FGF14 and different Nav channel isoforms with antigens that are known for being fixative sensitive (Mojumder et al., 2007; Tian et al., 2014). Using a panel of PanNav, Nav1.1, Nav1.2, and Nav1.6 antibodies in combination with FGF14 we were able to detect a sharp immunolabeling signal from FGF14 in all brain areas examined (Figures 8A–D, red channel), yet failed to detect signals from PanNav (Figure 8A), Nav1.1 (Figure 8B), or Nav1.2 (Figure 8C) (except for Nav1.2 in the cerebellum (Supplementary Figure 4C). Notably, though, highly detectable immunoreactivity was detected only for Nav1.6 found to co-localize with FGF14 at the AIS and with Caspr at the nodes of Ranvier (Caldwell et al., 2000) in various cortical regions (Figure 8D, Supplementary Figure 4D).

To further evaluate the strength of our findings, we examined the performance of another method used for AIS proteins detection (Rasband et al., 1999), which requires the brain to be directly immersed in 4% PFA for 30 min followed by an overnight sucrose cryoprotection, and compared it to the 1% formaldehyde and 0.5% MeOH method (Supplementary Figures 6, 7). The performance of the two methods varied depending on the combination of antibodies used and the brain regions examined. For instance, in the triple immunolabeling experiment of Supplementary Figure 6A, PanNav, spectrin and the mouse anti-PanNav clone K58/35 (AIS of CA1 pyramidal neurons) immunosignals were sharper with the direct 4% PFA immersed section method compared to the 1% formaldehyde and 0.5% MeOH condition (Supplementary Figure 6B), but two methods were comparable for the detection of FGF14 and Ankyrin-G (Supplementary Figures 6C,D). Though, the Nav1.6 immunosignal was superior when detected in sections fixed with 1% formaldehyde and 0.5% MeOH in double labeling experiments (Supplementary Figures 7A,B) and single labeling in different brain regions (Supplementary Figures 7C,D). Furthermore, we consistently found that the tissue integrity was much more preserved in 1% formaldehyde and 0.5% MeOH compared to 4% PFA brief immersion in which tissue tearing was commonly observed and laminar structures were not fully intact (Supplementary Figure 7).

### Option C: validation of 1% formaldehyde ± 0.5% MeOH in live tissue preparation

**Figure 9:**
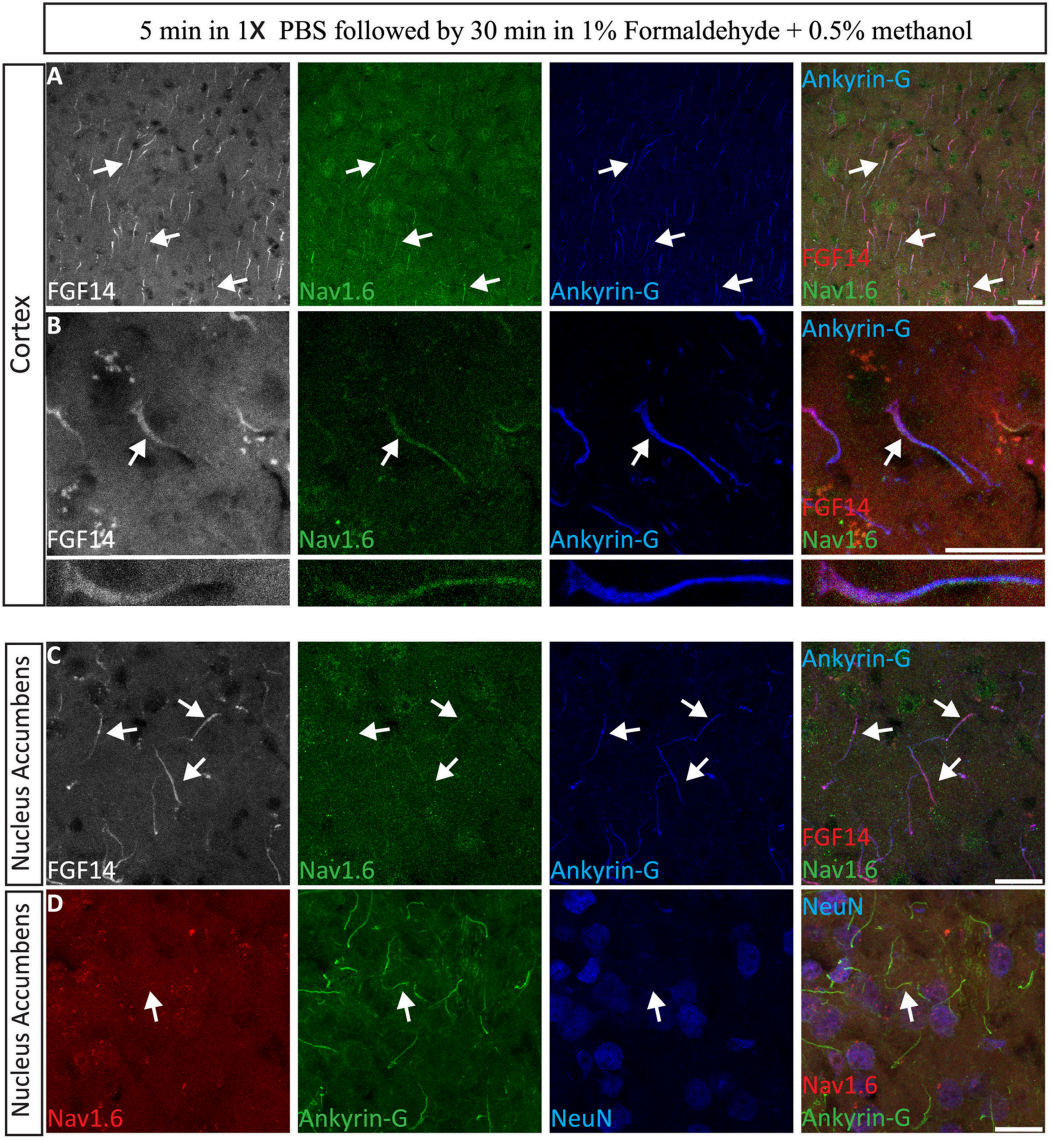
**Evaluation of the 1% formaldehyde + 0.5% MeOH fixation method for ***post-hoc*** analysis in acute brain slices**. **(A–C)** The gray channel represents FGF14 immunoreactivity visualized with an Alexa 568-conjugated secondary antibody. The green channel represents Nav1.6 (Alomone Labs) visualized with an Alexa 488-conjugated secondary antibody, and the blue represents Ankyrin-G (NeuroMab, catalog number 75-146) visualized with an Alexa 647-conjugated secondary antibody in the cortex at low in **(A,C)** and high in **(B)** magnification. Images in **(A,B)** are from the cortex while images in **(C)** are taken from the NAc. **(D)** The red channel represents Nav1.6 (Alomone Labs) immunoreactivity visualized with an Alexa 568-conjugated secondary antibody. The green channel represents Ankyrin-G (NeuroMab, catalog number 75–146) visualized with an Alexa 488-conjugated secondary antibody and the blue represents NeuN (visualized with an Alexa 647-conjugated secondary antibody) in the NAc. Arrows show FGF14 and/or Nav1.6 signals at the axon initial segment (AIS). NAc, nucleus accumbens. Scale bars represent 20 μm.

The immunolabeling of proteins in acute brain slice preparations, typically used for functional studies (i.e., electrophysiology), is a desirable technique to correlate neuronal activity outcomes with protein expression profiles. To evaluate the performance of our 1% formaldehyde + 0.5% MeOH protocol in acute brain slice preparations (in which animals were either promptly decapitated or perfused with physiological saline solution) ~300 μm thick freshly prepared acute brain slices were transferred to a recovery chamber for 2 h, immersed in 1% formaldehyde + 0.5% MeOH for 30 min, and kept in 20–30% sucrose overnight (Table 3). The tissue was then sectioned the following day and immunostaining performed. As shown in Figure 9A, FGF14 was found co-localized with Nav1.6 and Ankyrin-G in the cortex and the NAc (Figure 9B), indicating that this fixation protocol provides a highly sensitive tool for the detection of FGF14 and Nav1.6 channels in fine sub-cellular structures in the brain (Figures 9C,D).

## Discussion

We specifically conducted this study to: i. overcome previous limits in detecting FGF14 immunolabeling at the AIS while maintaining well-preserved cell and tissue morphology; ii. validate our staining approach in different brain regions using multiple markers; iii. further optimize the protocol for well-known fixative-sensitive proteins such as Nav1.6, a key binding partner of FGF14; and iv. generate a protocol suitable for *post-hoc* IHC following functional electrophysiological studies. Our overall finding is that the fixation procedure is the key step in successfully detecting fluorescent immunolabeling signals (of any protein) and that meticulous trial-and-error optimizations of the fixation step to better expose the antigen can reveal the subcellular distribution of analytes that were otherwise undetectable with traditional protocols.

Proper fixation is critical for unmasking certain antigens (Schneider Gasser et al., 2006; Christensen et al., 2014; Lorenzo et al., 2014) and optimization of this step in IHC protocols can significantly impact antibody detection specificity. Inappropriate fixation can also lead to a non-specific signal and high background to noise ratio diminishing the power of immunoprobes (Schneider Gasser et al., 2006; Fritschy, 2008).

Every fixation protocol, though, has advantages and pitfalls. For instance, the fresh-frozen tissue approach provides preservation of chemical antigenicity, at least for some fixative-sensitive proteins in tightly organized cellular microdomains. However, it bears limited results for overall tissue integrity and cellular architecture (Niki et al., 2004; Lajtha et al., 2007). The other general method, the fixed tissue approach, relies basically on the formaldehyde chemistry. Formaldehyde and its derivative para-formaldehyde (PFA) are crosslinking agents that chemically modify the free amino groups in amino acid chains. PFA, delivered in the animal through the vasculature, is one of the most widely used fixatives as it provides a simple and accessible method for studying cellular localization and expression patterns of given analytes and morphological studies at the cellular and subcellular level (Stradleigh and Ishida, 2015). Formaldehyde does have some drawbacks though, the major being epitope masking (Hoetelmans et al., 2001). Studies have recognized this problem for IHC targeting neurotransmitters (Stradleigh and Ishida, 2015), myelin (Christensen et al., 2014), synapses (Schneider Gasser et al., 2006; Lorenzo et al., 2014) and the AIS (Tian et al., 2014). Coagulant fixatives such as methanol and acetone are yet another class of fixatives. These compounds can lead to poor tissue preservation and limited detection of subcellular proteins. They are known to cause dehydration and extraction of membrane lipids (Bancroft and Stevens, 1990; Hoetelmans et al., 2001; Al-Mulla, 2011) leading to tissue shrinkage and tearing. However, when compared to crosslinking agents, coagulants perform better for epitope antigenicity since coagulants do not interact covalently with amino acid residues preventing major changes in the secondary and tertiary structure of proteins (Al-Mulla, 2011).

The level of detail achieved by this investigation identifies two ideal fixation conditions for triple IHC experiments targeting FGF14 that include mixing different classes of fixatives: 1% formaldehyde + 0.5% MeOH, and 4% PFA + cold acetone. In addition, although some methods failed to detect FGF14 immunoreactivity, they provided satisfactory detection of other antigens. For example, Optimal Fix™ perfusion was ideal for calbindin staining in the cerebellum. Likewise, the 4% PFA perfusion was optimal for parvalbumin detection in individual neuron soma and dendritic arborization but limited for FGF14 detection. Similar suboptimal results were obtained for calbindin and FGF14 staining following 1% PFA fixation.

Interestingly, we found significant variations in the requirements for fixation depending on brain areas. For example, the 4% PFA + 7 min acetone condition worked quite efficiently for detecting FGF14 in the cortex and the hippocampal DG region. However, these signals were harder to detect in the hippocampal CA1 region where principal cells are densely packed in a laminated structure that might slow diffusion and penetration of fixative agents (Rho et al., 2010). Indeed, FGF14 immunoreactivity in the CA1 region could be detected, but only using a combination of 1% formaldehyde and 0.5% MeOH, likely because of the methanol effect on antigen unmasking.

Using these improved IHC methods we have identified FGF14 as a component of the AIS in parvalbumin, calbindin and NeuN positive cells in multiple brain regions. We also succeeded in visualizing FGF14 in migratory neuroblasts, doublecortin positive cells and Sox2^+^ neuronal stem cells, as shown in other studies from our laboratory (Alshammari et al., 2015). However, we were unable to detect FGF14 signals in BrdU^+^ cells, possibly because BrdU processing requires an intense acidic treatment that might alter antigen conformation or exposure.

Previous studies have shown that FGF14 regulates different Nav channel isoforms. FGF14 co-localizes with PanNav channels in primary culture neurons (Lou et al., 2005; Laezza et al., 2007, 2009; Shavkunov et al., 2013), in hippocampal and para-hippocampal regions (Shavkunov et al., 2013), and in the axon initial segment of cerebellar granule cells (Diwakar et al., 2009). Our studies confirm, and expand upon, these findings in brain areas such as the cortex and cerebellum. Our studies also confirmed the high sensitivity of different Nav channel isoforms to fixation and tissue preparation as previously reported in the mammalian retina (Mojumder et al., 2007). Among all the Nav isoforms, we were most able to greatly optimize Nav1.6 staining. For this Nav isoform, fixation with 1% formaldehyde + 0.5% MeOH leads to optimal detection in double and triple staining experimental sets including FGF14. We confirmed Nav1.6 staining using two different antibodies, a polyclonal anti-rabbit (Alomone Labs) and a monoclonal mouse anti-Nav 1.6 antibody (NeuroMab). Further validation was provided using various tissue preparations leading to a high signal overlap between FGF14 and Nav1.6 in snap-frozen, perfusion-fixed and acute brain slices. The Nav1.1 immunosignal at the AIS was not well defined in tissue fixed with 1% formaldehyde + 0.5% MeOH, although some immunoreactivity in the soma was detected. This result may reflect a true sub-cellular specific distribution of Nav1.1 with a stronger signal in the soma than at the AIS (Gong et al., 1999; Caldwell and Levinson, 2004; Kalume et al., 2007) or it might just indicate that further optimization is still needed for this antigen. As for Nav1.2, we detected Nav1.2 signal in the AIS of cells in the cerebellum where it co-localized with FGF14 (data not shown). Previous studies in the retina have shown that PanNav labeling is extremely sensitive to fixation (Mojumder et al., 2007). Our studies confirmed this report showing that immunolabeling of all Nav channels with PanNav antibodies (raised against conserved intracellular epitopes) was limited to 1% formaldehyde + 0.5% MeOH fixed tissue or fresh-frozen tissue (Shavkunov et al., 2013). Finally, we compared the 1% formaldehyde + 0.5% MeOH with the 4% PFA whole-brain immersion method that has been previously used to examine AIS components (Rasband et al., 1999). Our study indicates that the two methods led to variable results with the 4% PFA immersion method being superior for the detection of mouse anti-PanNav (clone K58/35) and βIV-spectrin, but comparable or inferior to the 1% formaldehyde + 0.5% MeOH method in the detection of Ankyrin-G, FGF14, and NeuN or Nav1.6, respectively. Overall, though, across all immunostainings the 1% formaldehyde + 0.5% MeOH method resulted in much better tissue integrity compared to the 4% PFA immersion method in which tissue tearing was commonly observed.

## Conclusion and future directions

We have designed two ideal protocols for detecting FGF14 alone or in combination with Nav1.6 at the AIS, in double and triple IHC experiments in multiple brain regions and cell types. One successful method includes a modified version of the routinely used 4% PFA perfusion fixation process. The second one consists of 1% formaldehyde + 0.5% MeOH, a useful approach for *post-hoc* IHC following electrophysiological studies. The level of sensitivity of our methods provides new tools for quantitative profiling of proteins in fixative-resistant cellular microdomains such as the AIS, the node of Ranvier and pre and post-synaptic compartments. Given the improved signal-to-noise ratio and detection power, our methods will also improve the performance of fluorescence-based image analysis including tracking, segmentation, and development of algorithms for geometrical descriptors in cell reconstruction and classification studies (Labate et al., 2014; Ozcan et al., 2015).

## Technical comments

Lightly fixed tissue tends to be thinner, fragile and more susceptible to tearing. Direct adhering of tissue slices to positively charged glass slides is a solution to this problem. Importantly, a successful approach requires the tissue to be <20 μm thick to prevent tissue fall off during the staining procedure (i.e., permeabilization, washing, etc.) and to favor tissue adherence to the charged side of the glass slide. Another technical spotlight is the auto-fluorescence and high background noise associated with cross-linking fixative agents (Stradleigh and Ishida, 2015) which should always be taken into consideration especially when optimizing uncharacterized antibodies. Thus, using tissue that has been properly prepared, stored and kept under controlled humidity conditions during the staining, and processed with the most appropriate fixative agent/s (such as varying concentrations of PFA accompanied by light fixation with cold acetone and/or methanol) is one of the key ingredients to successful IHC (Schneider Gasser et al., 2006).

## Author contributions

MA and TA contributed to the design of the work, the acquisition, and interpretation of the data. MA and TA performed tissue cryo-sectioning, immunohistochemistry, confocal images; MA processed mouse tissue, supervised, maintained, and genotyped the animal colony. FL contributed to the design of the work, provided resources, and intellectual support. MA, TA, and FL wrote manuscript; all experiments were performed in FL laboratory.

### Conflict of interest statement

The authors declare that the research was conducted in the absence of any commercial or financial relationships that could be construed as a potential conflict of interest.

## Supplementary material

Supplementary Figure 1 **Validation of FGF14 staining using ***Fgf14***^−∕−^ mouse tissue fresh-frozen sections**. Immunofluorescent staining of a representative sagittal section of mouse brain showing FGF14 immunoreactivity (gray and red) in the cortex from *Fgf14*^+∕+^ and *Fgf14*^−∕−^ mice in combination with PanNav in green channel (Alomone Labs). The arrows show co-localization of FGF14 and PanNav at axon initial segment (AIS) in *Fgf14*^+∕+^, but not *Fgf14*^−∕−^ tissue. Scale bars represent 20 μm.

Supplementary Figure 2 **Validation of FGF14 staining using ***Fgf14***^−∕−^ mouse tissue using 4% PFA with light acetone fixation**. Immunofluorescent staining of a representative sagittal section of mouse brain showing FGF14 immunoreactivity (gray and red) in the PFC from *Fgf14*^+∕+^ and *Fgf14*^−∕−^ mice in combination with Topro3 (blue) nuclear staining in blue. The arrows show FGF14 staining at the axon initial segment (AIS) in *Fgf14*^+∕+^, but not *Fgf14*^−∕−^ tissue. Scale bars represent 20 μm.

Supplementary Figure 3 **Validation of FGF14 staining using ***Fgf14***^−∕−^ mouse tissue using 1% formaldehyde + 0.5% MeOH followed by light acetone fixation**. Immunofluorescent staining of a representative sagittal section of mouse brain showing FGF14 immunoreactivity (gray and red) in the CA1 hippocampal region from *Fgf14*^+∕+^ and *Fgf14*^−∕−^ mice in combination with Ankyrin-G (blue) and NeuN (green). The arrows show co-localization of FGF14 and Ankyrin-G at axon initial segment (AIS) in *Fgf14*^+∕+^, but not *Fgf14*^−∕−^ tissue. Scale bars represent 20 μm.

Supplementary Figure 4 **FGF14 and Nav1 α-subunit-specific antibodies staining perfusion-fixed tissue preparations (1% formaldehyde +0.5% MeOH) followed by light acetone fixation. (A–C)** Representative immunostaining of FGF14 (gray and red) in combination with PanNav (Alomone Labs) in the cortex A, Nav1.2 (NeuroMab) in the subiculum **(B)**, and cerebellum **(C)**, all shown in green. Topro3 nuclear staining from sagittal sections of *Fgf14*^+∕+^ mice is shown in blue. Arrows show localization of FGF14 at axon initial segment (AIS); note co-localization with Nav1.2 only in the cerebellum **(C). (D)** Green channel shows Nav1.6 (Alomone Labs) clustering at the nodes of Ranvier (arrowheads) label with Caspr (red) in the cortical region. Scale bars represent 20 μm.

Supplementary Figure 5 **FGF14, Ankyrin-G, calbindin, and MAP2 staining using 4% PFA + acetone, and 1% formaldehyde + 0.5% MeOH fixation conditions. (A,B)** The gray and red channels represent FGF14 immunoreactivity visualized with an Alexa 568-conjugated secondary antibody. The green channel represents calbindin [gray and green in **(B)**] visualized with an Alexa 488-conjugated secondary in combination with MAP2 [Synaptic System; blue in **(A)**, gray and blue in **(B)**] visualized with an Alexa 647-conjugated secondary antibody using 4% PFA + acetone fixation from prefrontal cortex (PFC) and hippocampal CA1 regions in A, and 1% formaldehyde + 0.5% MeOH from the dentate gyrus in **(B). (C)** The green channel represents Ankyrin-G (NeuroMab, catalog number 75–146) visualized with an Alexa 647-conjugated secondary antibody. The red channel represents MAP2 immunoreactivity (Novus Biologicals) visualized with an Alexa 568-conjugated secondary antibody using 1% formaldehyde + 0.5% MeOH fixation. Arrows show localization of FGF14 or Ankyrin-G at axon initial segment (AIS). Scale bars represent 20 μm.

Supplementary Figure 6 **Comparing 1% formaldehyde + 0.5% MeOH to the 4% PFA immersion fixation to detect AIS components**. (**A**,**C**) represent 4% PFA immersion fixation (Scheme 1, Option B, forth column of Table 2). (**B**,**D**) represent 1% formaldehyde and 0.5% methanol fixation (Scheme 1, Option B, second column of Table 2). In (**A**,**B**) the gray and red channel show PanNav clone K58/35 visualized with an Alexa 568-conjugated secondary antibody in combination with βIV-spectrin in the green channel (visualized with an Alexa 488-conjugated secondary antibody) and NeuN in the blue channel. Arrows show co-localization between PanNav and βIV-spectrin at the AIS. In **(C,D)** the gray and red channel show FGF14 visualized with an Alexa 568-conjugated secondary antibody in combination with Ankyrin-G in the green channel (visualized with an Alexa 488-conjugated secondary antibody). Arrows show co-localization between FGF14 and Ankyrin-G at the AIS. Scale bars represent 20 μm.

Supplementary Figure 7 **Nav1.6 and PanNav detection using 1% formaldehyde + 0.5% MeOH and 4% PFA immersion fixation conditions. (A)** The gray and green channel show Nav1.6 (Alomone Labs) visualized with an Alexa 488-conjugated secondary antibody in combination with PanNav clone K58/35 in gray and red channel visualized with an Alexa 568-conjugated secondary antibody using 1% formaldehyde + 0.5% methanol fixation or 4% PFA immersion fixation in **(B)**. **(C,D)** The gray channel shows Nav1.6 in different brain regions using 1% formaldehyde + 0.5% methanol fixation or 4% PFA immersion fixation in **(D)**. Arrows show co-localization between Nav1.6 and PanNav at the AIS. Asterisks indicate tissue tearing visible in the 4% PFA immersion method. Scale bars represent 20, 40 μm in **(D)**.
