# Supplementary figures and images for: Improved Methods for Fluorescence Microscopy Detection of Macromolecules at the Axon Initial Segment
Source: Front Cell Neurosci. 2016 Feb 16;10:5. doi: 10.3389/fncel.2016.00005 (PMC4754416; doi:10.3389/fncel.2016.00005)

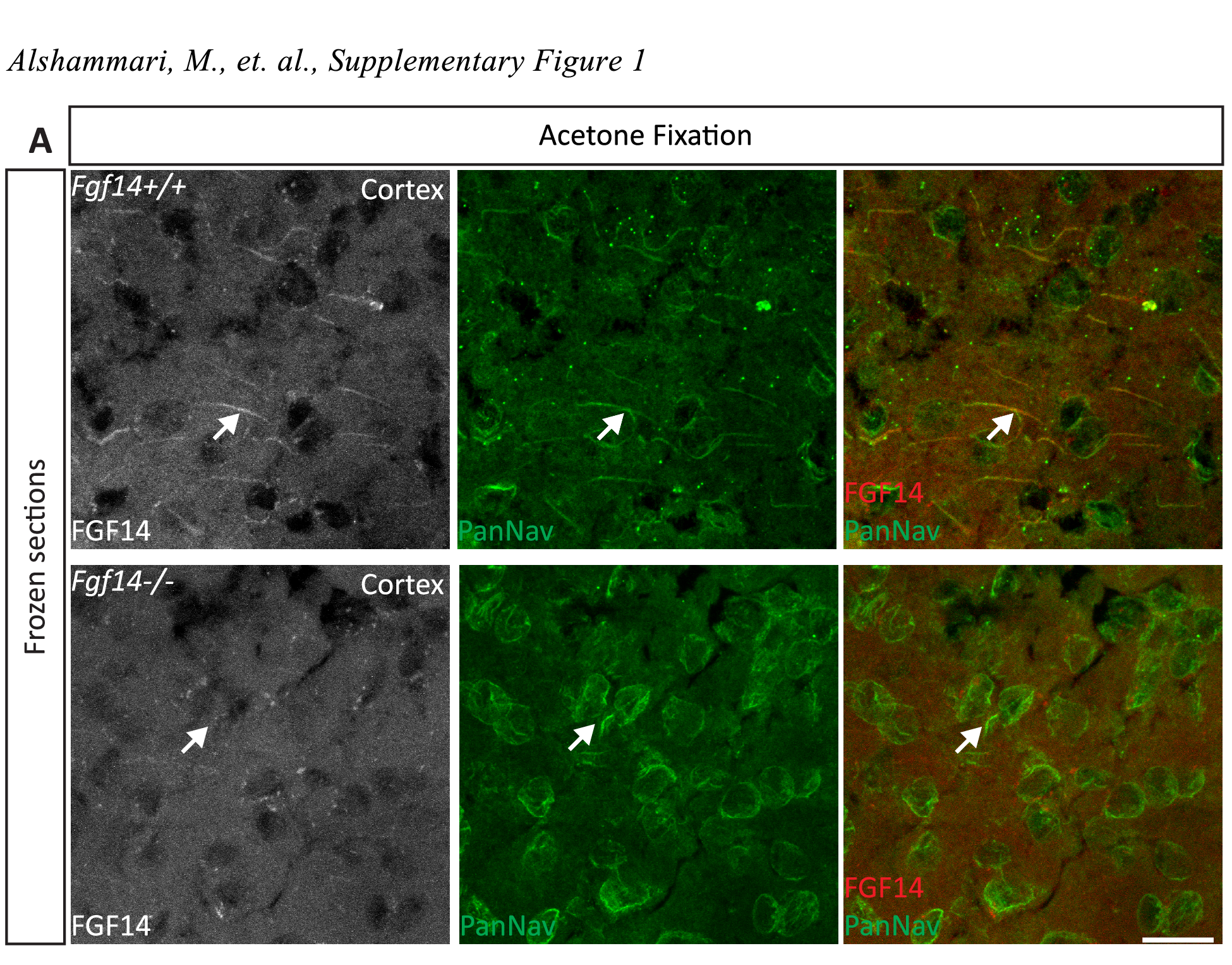

Supplement: Supplementary Figure 1 — Validation of FGF14 staining using Fgf14−∕− mouse tissue fresh-frozen sections. Immunofluorescent staining of a representative sagittal section of mouse brain showing FGF14 immunoreactivity (gray and red) in the cortex from Fgf14+∕+ and Fgf14−∕− mice in combination with PanNav in green channel (Alomone Labs). The arrows show co-localization of FGF14 and PanNav at axon initial segment (AIS) in Fgf14+∕+, but not Fgf14−∕− tissue. Scale bars represent 20 μm. [file Image1.TIF]

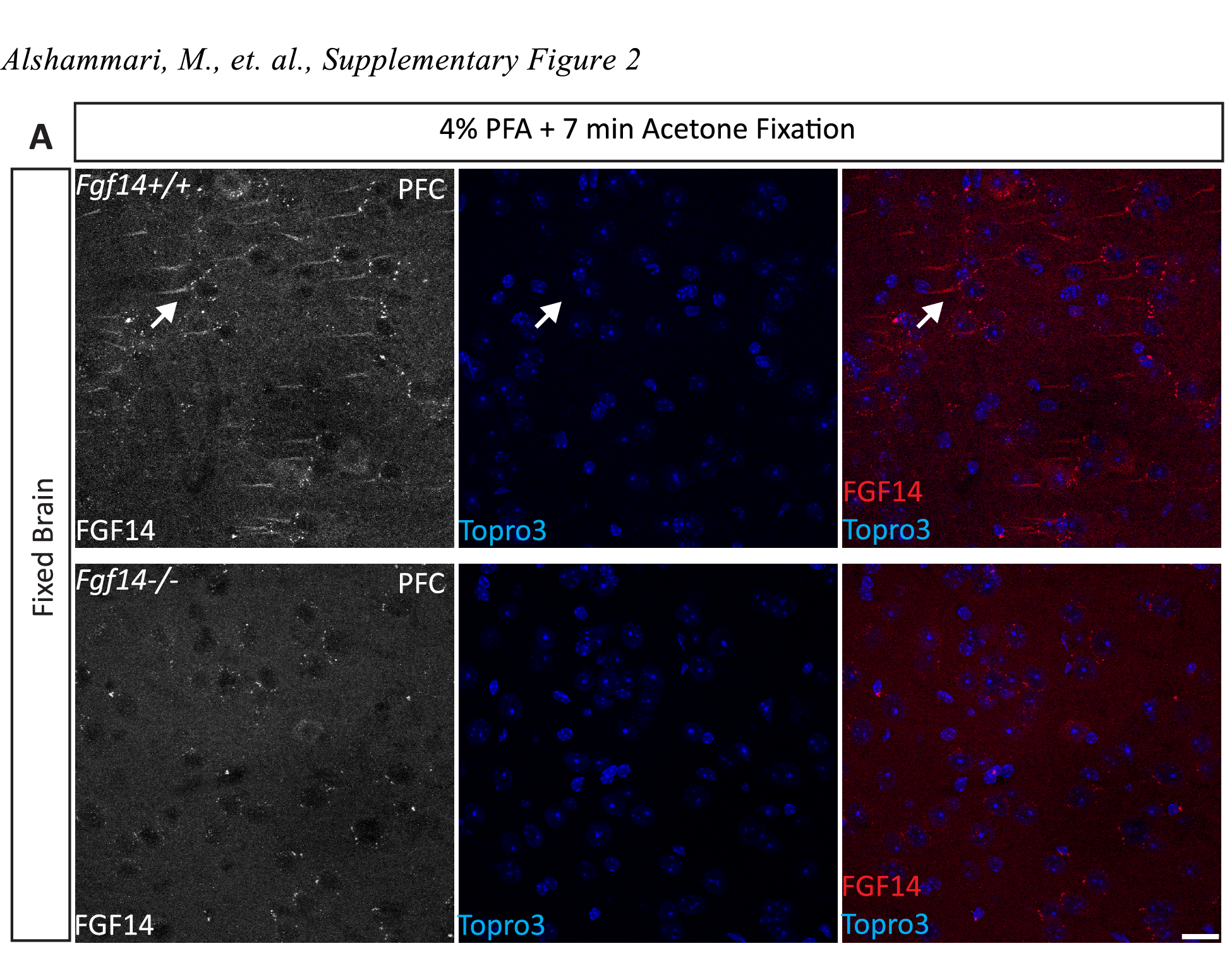

Supplement: Supplementary Figure 2 — Validation of FGF14 staining using Fgf14−∕− mouse tissue using 4% PFA with light acetone fixation. Immunofluorescent staining of a representative sagittal section of mouse brain showing FGF14 immunoreactivity (gray and red) in the PFC from Fgf14+∕+ and Fgf14−∕− mice in combination with Topro3 (blue) nuclear staining in blue. The arrows show FGF14 staining at the axon initial segment (AIS) in Fgf14+∕+, but not Fgf14−∕− tissue. Scale bars represent 20 μm. [file Image2.TIF]

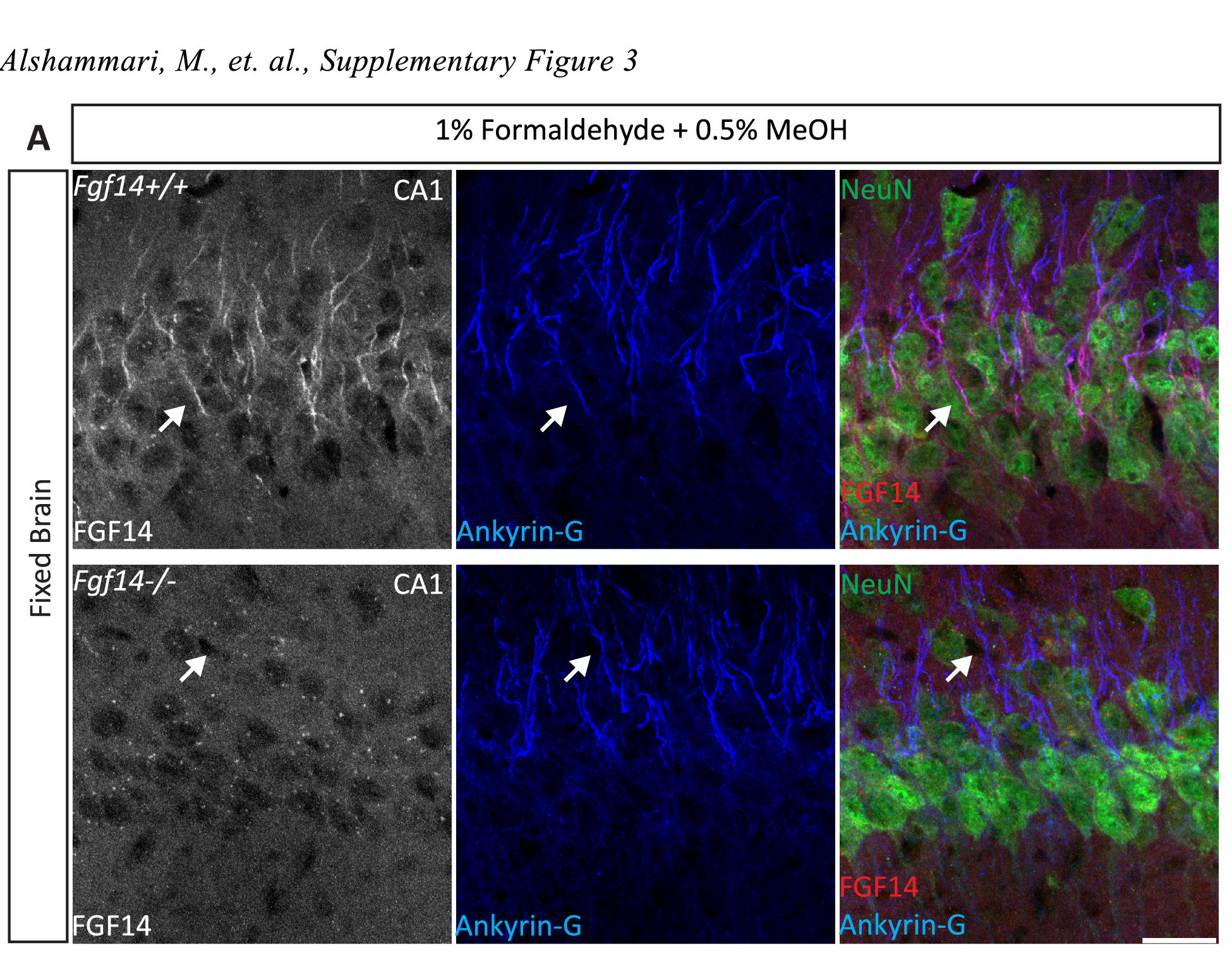

Supplement: Supplementary Figure 3 — Validation of FGF14 staining using Fgf14−∕− mouse tissue using 1% formaldehyde + 0.5% MeOH followed by light acetone fixation. Immunofluorescent staining of a representative sagittal section of mouse brain showing FGF14 immunoreactivity (gray and red) in the CA1 hippocampal region from Fgf14+∕+ and Fgf14−∕− mice in combination with Ankyrin-G (blue) and NeuN (green). The arrows show co-localization of FGF14 and Ankyrin-G at axon initial segment (AIS) in Fgf14+∕+, but not Fgf14−∕− tissue. Scale bars represent 20 μm. [file Image3.TIF]

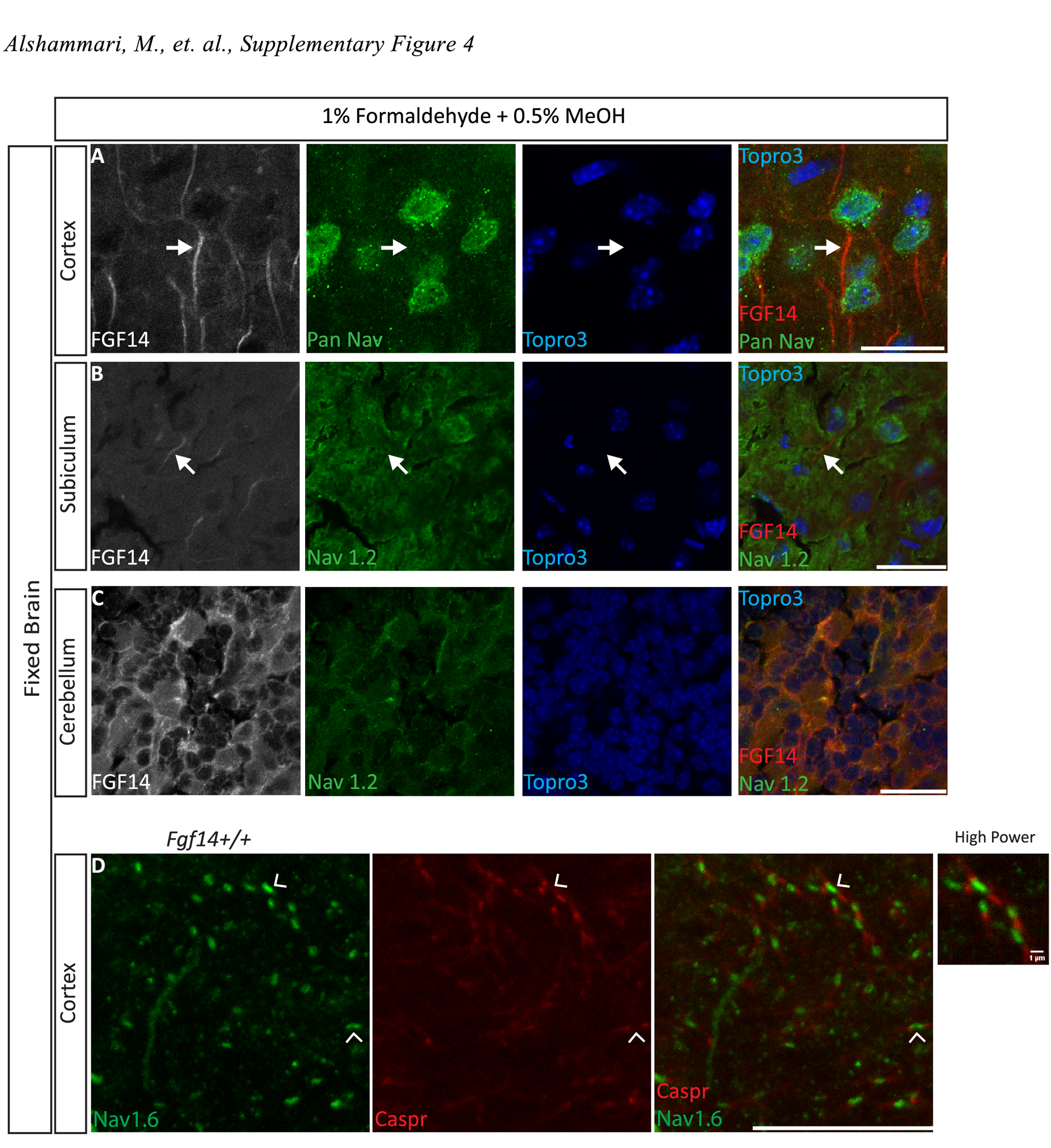

Supplement: Supplementary Figure 4 — FGF14 and Nav1 α-subunit-specific antibodies staining perfusion-fixed tissue preparations (1% formaldehyde +0.5% MeOH) followed by light acetone fixation. (A–C) Representative immunostaining of FGF14 (gray and red) in combination with PanNav (Alomone Labs) in the cortex A, Nav1.2 (NeuroMab) in the subiculum (B), and cerebellum (C), all shown in green. Topro3 nuclear staining from sagittal sections of Fgf14+∕+ mice is shown in blue. Arrows show localization of FGF14 at axon initial segment (AIS); note co-localization with Nav1.2 only in the cerebellum (C). (D) Green channel shows Nav1.6 (Alomone Labs) clustering at the nodes of Ranvier (arrowheads) label with Caspr (red) in the cortical region. Scale bars represent 20 μm. [file Image4.TIF]

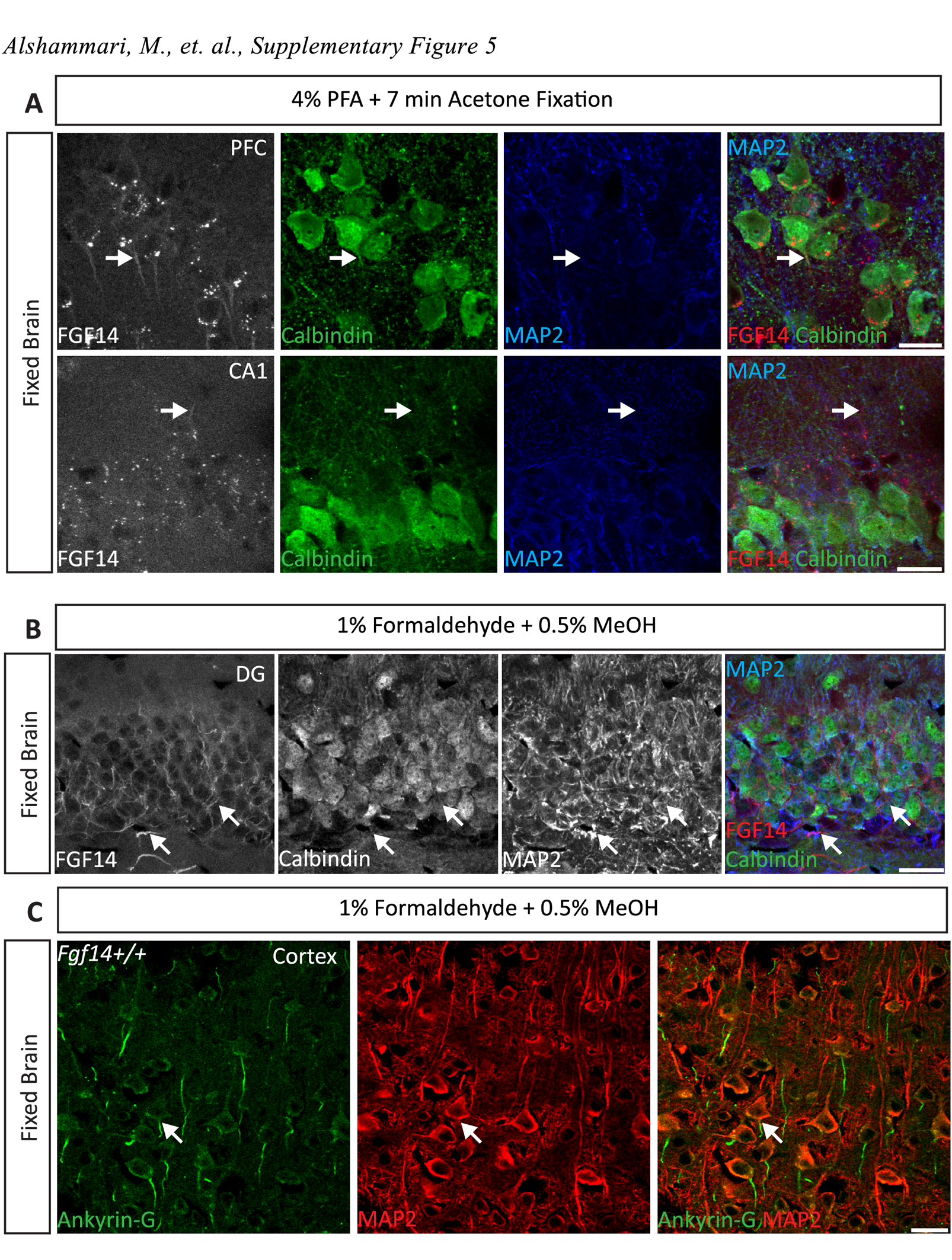

Supplement: Supplementary Figure 5 — FGF14, Ankyrin-G, calbindin, and MAP2 staining using 4% PFA + acetone, and 1% formaldehyde + 0.5% MeOH fixation conditions. (A,B) The gray and red channels represent FGF14 immunoreactivity visualized with an Alexa 568-conjugated secondary antibody. The green channel represents calbindin [gray and green in (B)] visualized with an Alexa 488-conjugated secondary in combination with MAP2 [Synaptic System; blue in (A), gray and blue in (B)] visualized with an Alexa 647-conjugated secondary antibody using 4% PFA + acetone fixation from prefrontal cortex (PFC) and hippocampal CA1 regions in A, and 1% formaldehyde + 0.5% MeOH from the dentate gyrus in (B). (C) The green channel represents Ankyrin-G (NeuroMab, catalog number 75–146) visualized with an Alexa 647-conjugated secondary antibody. The red channel represents MAP2 immunoreactivity (Novus Biologicals) visualized with an Alexa 568-conjugated secondary antibody using 1% formaldehyde + 0.5% MeOH fixation. Arrows show localization of FGF14 or Ankyrin-G at axon initial segment (AIS). Scale bars represent 20 μm. [file Image5.TIF]

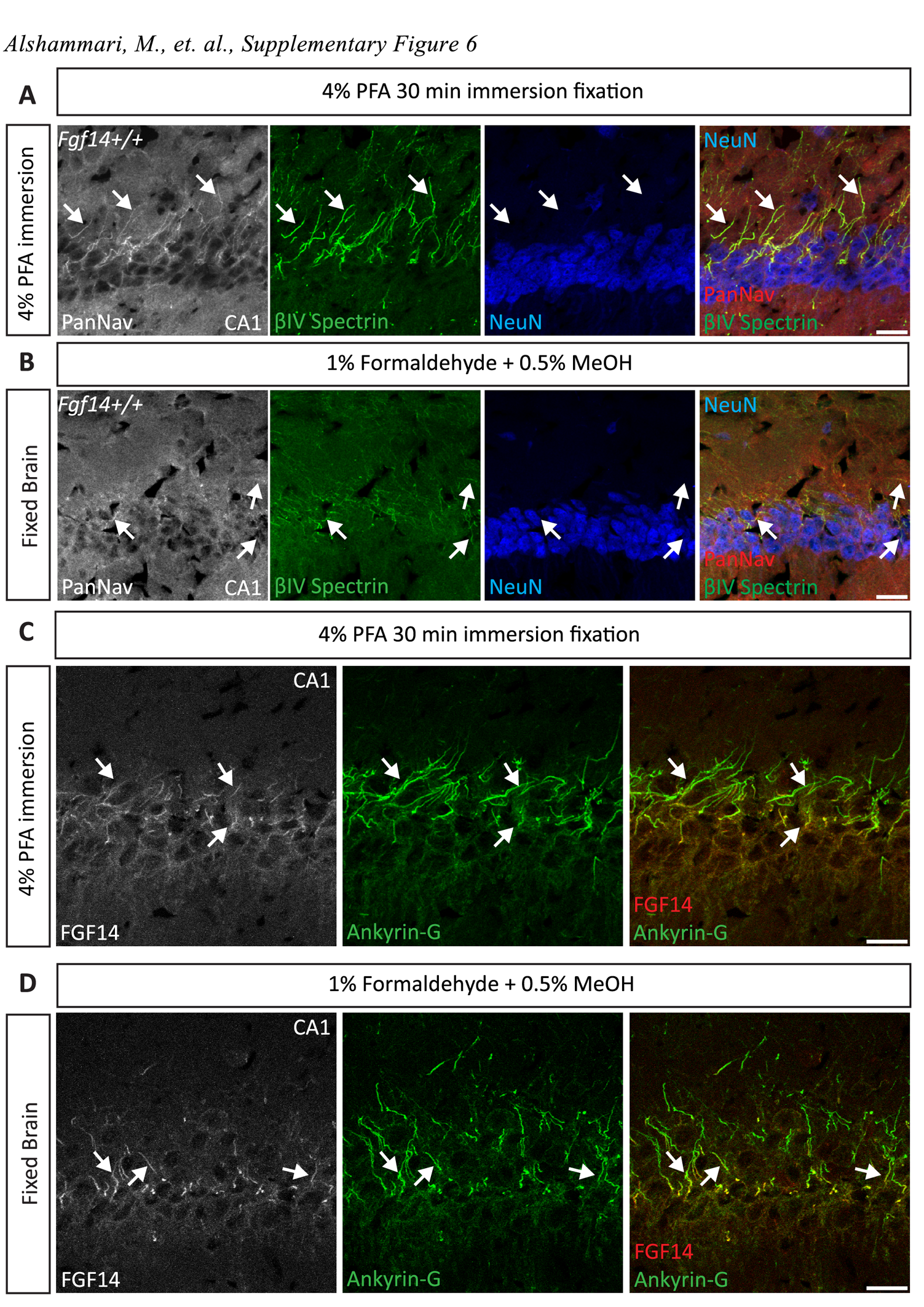

Supplement: Supplementary Figure 6 — Comparing 1% formaldehyde + 0.5% MeOH to the 4% PFA immersion fixation to detect AIS components. (A,C) represent 4% PFA immersion fixation (Scheme 1, Option B, forth column of Table 2). (B,D) represent 1% formaldehyde and 0.5% methanol fixation (Scheme 1, Option B, second column of Table 2). In (A,B) the gray and red channel show PanNav clone K58/35 visualized with an Alexa 568-conjugated secondary antibody in combination with βIV-spectrin in the green channel (visualized with an Alexa 488-conjugated secondary antibody) and NeuN in the blue channel. Arrows show co-localization between PanNav and βIV-spectrin at the AIS. In (C,D) the gray and red channel show FGF14 visualized with an Alexa 568-conjugated secondary antibody in combination with Ankyrin-G in the green channel (visualized with an Alexa 488-conjugated secondary antibody). Arrows show co-localization between FGF14 and Ankyrin-G at the AIS. Scale bars represent 20 μm. [file Image6.TIF]

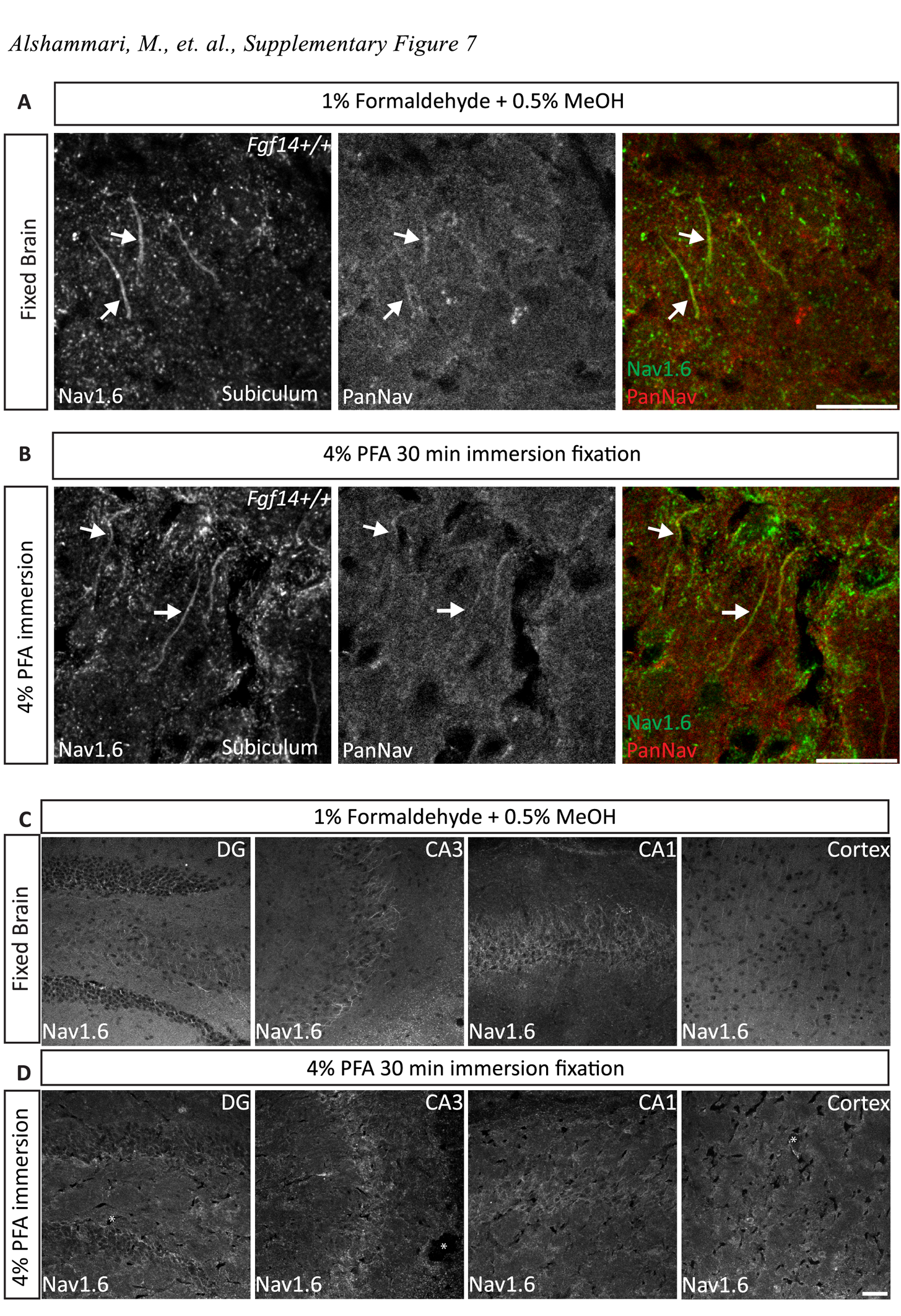

Supplement: Supplementary Figure 7 — Nav1.6 and PanNav detection using 1% formaldehyde + 0.5% MeOH and 4% PFA immersion fixation conditions. (A) The gray and green channel show Nav1.6 (Alomone Labs) visualized with an Alexa 488-conjugated secondary antibody in combination with PanNav clone K58/35 in gray and red channel visualized with an Alexa 568-conjugated secondary antibody using 1% formaldehyde + 0.5% methanol fixation or 4% PFA immersion fixation in (B). (C,D) The gray channel shows Nav1.6 in different brain regions using 1% formaldehyde + 0.5% methanol fixation or 4% PFA immersion fixation in (D). Arrows show co-localization between Nav1.6 and PanNav at the AIS. Asterisks indicate tissue tearing visible in the 4% PFA immersion method. Scale bars represent 20, 40 μm in (D). [file Image7.TIF]
